# Determinants of Short Interbirth Interval among Reproductive Age Mothers in Arba Minch District, Ethiopia

**DOI:** 10.1155/2016/6072437

**Published:** 2016-04-27

**Authors:** Desta Hailu, Teklemariam Gulte

**Affiliations:** ^1^Department of Nursing, College of Medicine & Health Sciences, Arba Minch University, P.O. Box 21, Arba Minch, Ethiopia; ^2^Department of Midwifery, College of Medicine & Health Sciences, Arba Minch University, P.O. Box 21, Arba Minch, Ethiopia

## Abstract

*Background*. One of the key strategies to reduce fertility and promote the health status of mothers and their children is adhering to optimal birth spacing. However, women still have shorter birth intervals and studies addressing their determinants were scarce. The objective of this study, therefore, was to assess determinants of birth interval among women who had at least two consecutive live births.* Methods*. Case control study was conducted from February to April 2014. Cases were women with short birth intervals (<3 years), whereas controls were women having history of optimal birth intervals (3 to 5 years). Bivariate and multivariable analyses were performed.* Result*. Having no formal education (AOR = 2.36, 95% CL: [1.23–4.52]), duration of breast feeding for less than 24 months (AOR: 66.03, 95% CI; [34.60–126]), preceding child being female (AOR: 5.73, 95% CI; [3.18–10.310]), modern contraceptive use (AOR: 2.79, 95% CI: [1.58–4.940]), and poor wealth index (AOR: 4.89, 95% CI; [1.81–13.25]) of respondents were independent predictors of short birth interval.* Conclusion*. In equalities in education, duration of breast feeding, sex of the preceding child, contraceptive method use, and wealth index were markers of unequal distribution of inter birth intervals. Thus, to optimize birth spacing, strategies of providing information, education and communication targeting predictor variables should be improved.

## 1. Introduction 

Although rates of population increase are now much slower in the developed world than in the developing countries, the world's population has been growing rapidly in recent decades. Much of this increase comes from high-fertility Africa countries. Sub-Saharan Africa and Asia particularly have population growth rates that are outpacing their economic growth [1]. Ethiopia, the second most populous country in Africa next to Nigeria, has a total population of 79.8 million [2]. The population of the country has significantly increased as compared to the previous consecutive censuses periods (1984 and 1994) when it was about 40 and 53.5 million, respectively [3].

Like many other Sub-Saharan African countries, fertility rate is profoundly higher in Ethiopia (4.8) as compared to the global figure (2.69) [4, 5]. While the average lifetime fertility has declined in the past 15 years, from a 1990 level of 6.4 births per woman down to 4.8 births in 2011, rural women still have an average of three more births per woman compared to women in urban areas (5.5 and 2.6, resp.) [2, 5]. Even with fertility decline, the population is still growing at an annual rate of 2.6%.

Birth interval, defined as the number of months between the birth of the child under study and the immediately preceding birth to the mother, has a critical effect on the population size and the health status of the mother and her child [6, 7]. Evidence showed that a relationship prevails between shorter birth intervals and high infant and child mortality [8, 9]. It was also indicated that short interbirth intervals have been linked to increased risk for preterm birth, low birth weight, being small for gestational age (SGA), labor dystocia, and maternal morbidity and mortality [5, 9]. Short birth intervals are also associated with high rates of premature rupture of membranes, third-trimester bleeding, anemia, and puerperal endometritis which place women at greater risk of hemorrhage, the primary cause of maternal death [4, 9].

Beyond the health and survival implications of high levels of closely spaced and unintended births, high fertility rates accelerate population growth and undermining development efforts across all sectors. Closely spaced births have a potentially devastating impact on both the individual and the society. This pattern, combined with high levels of unplanned fertility, makes it difficult for women to become productive members of society, thereby limiting their contribution to economic development [9].

While it is difficult to pinpoint the exact reasons, it has been established that the undesirable consequences of shorter interbirth intervals on perinatal, infant, and child survival and maternal mortality have been mainly attributed to maternal nutritional depletion syndrome [10].

Sibling competition increases in the situation of shorter interbirth intervals. It is argued that when a newborn comes, it is likely that the family will invest more of its limited resources in the form of care to the newborn and the other children are more likely to suffer or merely receive inadequate share of the resources distributed among siblings [9].

On the other hand, optimal birth spacing yields the greatest health, social, and economic benefits for the family [8]. Although previous research findings advocate an interval length of 2 years between two consecutive births for a better maternal and child health [8], recent evidence showed that births should be spaced at three to five years apart to ensure maximum health benefits for mothers, newborns, and older children. However, too long birth intervals (>5 years) are associated with increased risk of complications such as preeclampsia and eclampsia as the mother loses protective effect from the previous pregnancy [8, 9].

The Federal Ministry of Health (FMOH), reproductive health department, and health bureaus of respective regions have made concerted effort to reduce fertility. They have been applying multipronged approaches at local and national levels, fostering an enabling environment for family planning being at the heart of a variety of important strategies to address optimal birth spacing, and decreasing fertility [2].

Women in developing countries, however, still have shorter birth intervals than they would prefer [4]. In Ethiopia like many other Sub-Saharan African countries, fertility, maternal mortality, and child mortality are still high. Recent estimates showed that the country still experiences higher rates of maternal, neonatal, and infant mortality of 767/100,000, 37/1000, and 59 per 1000 live births, respectively [5]. However, little is known about the current level of birth spacing and determinant factors in Ethiopia and particularly in the study area. Thus, understanding the level of birth interval and factors influencing birth spacing is critical for countries like Ethiopia with a population policy aiming at reducing fertility. This study therefore aims to fill this gap by assessing the current level of interbirth interval and its determinant factors among rural child-bearing age women who gave birth in the five years preceding to this survey in Arba Minch Zuria Woreda, SNNP, Ethiopia.

## 2. Significance of the Study

In Ethiopia like many other Sub-Saharan African countries, fertility and maternal and child mortality are still high. Thus, acquiring research based knowledge of these problems is key step in undertaking appropriate intervention.

Therefore, the finding of this study would be helpful in reminding local and possibly nationwide policy makers of how fertility and birth spacing situation looks like in the study area and to design appropriate strategies for encouraging greater use of optimal birth spacing and thereby ensuring further declines in fertility and maternal and child mortality. It is also hoped that the result of this research will be an input for regional and district health care planners and program managers in designing site specific and scientifically sound interventions to address the gap in the utilization of family planning and optimal birth spacing.

In addition, the result will provide important information to local health care providers, community based health extension workers, civil organizations, and facility managers to design appropriate interventions suitable to their clients at facility and community level which can play an indispensable role in convincing mothers to optimize their birth interval in the district.

Finally, it is also expected to provide baseline information for further robust follow-up studies.

## 3. Literature Review

### 3.1. Birth Spacing Practice

Birth spacing is an important maternal and child health intervention. Studies have confirmed that healthy pregnancy timing and spacing are important interventions to improve infant, child, and maternal health. Although every pregnancy carries a risk of maternal death or morbidity, some pregnancies are at higher risk than others [11].

Recommendations for birth spacing made by international organizations were based on information that was available several years ago. Previous evidences by the World Health Organization (WHO) and other international organizations recommended that waiting for at least 2-3 years between pregnancies can reduce infant and child mortality and promote maternal health [8]. However, recent studies supported by the United States Agency for International Development (USAID) and World health organization (WHO) have suggested that, after a live birth, the recommended interval before attempting to have the next child is 3–5 years in order to reduce the risk of adverse maternal, perinatal, and infant outcomes [8, 9, 12].

According to the study done in Saudi Arabia, the mean duration of interbirth interval was reported to be 2.38 years [13]. Another study done in Babol, Northern Iran, revealed that the mean birth interval was 61 ± 25.7 months. In this study, 3.8%, 41.7%, and 28% of the respondents had a birth interval of <2, 4-5, and ≥6 years, respectively [14].

Similar Study from Rufiji, Tanzania, showed that the median interbirth interval was found to be 33.4 months. The same study revealed that 48.4% of the respondents had a birth interval below the WHO recommended minimum duration of 36 months [15].

Community based cross-sectional study conducted in Urban Saudi Arabia showed that the median duration of birth interval was 33.5 months. In this study, about a quarter of women had mean birth interval of <2 years while 35.1% had waited between 2 and 3 years [16].

Similarly, study from Ethiopia indicated that the majority (57%) of women were practicing short birth interval length (<3 years) with the median birth interval of 33 months [17].

### 3.2. Determinants of Birth Spacing

#### 3.2.1. Sociodemographic Factors

Evidences from different studies have stressed that the role of sociodemographic, economic, and birth history was significant in influencing interbirth intervals among child-bearing age mothers [11, 17, 18]. In this regard, good examples which have been repeatedly examined as determinants of length of birth interval like age of mother at last delivery, occupational status of the mother, marital status of the mother, educational status of the mother, religion of the mother, place of residence, monthly income, husband's educational status, husband's occupational status, sex of the index child, survival status of the index child, parity, duration of breast-feeding, and modern contraception use have been repeatedly examined as determinants of health care use.

According to health facility based cross-sectional study done among multiparous women in Babol, Northern Iran, age of the mother was a major determinant of birth interval. It indicated that as age increases birth interval decreases. Among the study participants, half (50%) of the women aged <20 years old and 0.9% of women ≥35 years old had a birth interval of <2 years, while 42.9% of the mothers aged ≥35 years old had a birth interval of ≥6 years, which increased with increasing maternal age, while the birth interval of <2 years decreased with increasing maternal age (*p* < 0.05) [14]. Other cross-sectional studies from Saudi Arabia, Denmark, Jordan, Nepal, and Pakistan showed similar findings (*p* < 0.05) [18–22].

Findings from African countries showed similar evidence. According to study done in Tanzania, maternal age was inversely related with nonadherence to the recommended minimum length between two live births. The proportion of interbirth intervals that were poorly spaced was highest (76%) among youngest (15–19) women and declined rapidly with increasing age to as low as 30% among the oldest (45–49) women (*p* < 0.001) [15].

In line with the above findings, report from Ethiopian DHS 2011 showed that young maternal age had significant association with birth interval. The median birth interval increases with age, ranging from 28.5 months for births to women aged 15–19 to 38.7 months for births to women aged 40–49 months [5].

Female education also is found to be a strong predictor of Birth intervals. Study done in Nepal indicated that level of education has shown strong statistical association with birth interval. Women with educational level of secondary stage and above were more likely to have longer birth intervals than those with elementary or no formal education [18]. Another study done in Jordan revealed that longer birth interval was independently predicted by woman's higher education [21]. Similar studies from Saudi Arabia, Jordan, and Pakistan indicated that maternal education had a positive linear relationship with birth intervals [13, 21, 22].

Study done in southern Ethiopia showed that maternal education has protective effect for short birth interval in which women who had no formal education were 1.9 times more likely to have short birth interval practice as compared to those who had formal education (AOR 1.89, 95% CI (1.15, 3.37)) [23]. Evidence from Ethiopian DHS 2011 indicated similar finding [5].

Regarding the occupation of respondents, study from Nepal, indicated that occupation of mothers found to be significant predictor of birth interval. Working mothers were more likely to have longer birth intervals than house wife mothers (*p* < 0.05) [18]. Study from Iran and Tanzania showed similar findings [24, 25]. Another study from Saudi indicated that Employment status was significantly related to birth interval. The optimum birth interval of 3–5 years was more frequently observed among women who were employed 171 (39.2%) than among home-makers 143 (32.7%) (*p* < 0.05) [19].

According to the study done in Nepal, occupations of husbands were significantly associated with birth intervals. Women whose husbands were engaged in agriculture had longer birth intervals as compared to those working in business and cottage industry [18]. Another study done in Southern Ethiopia showed that women whose husbands were students were found to be significant predictors of short birth interval [17].

It has also been found that the place where a woman resides influences the length of intervals between their births. Study in Nepal and Tanzania indicated that women residing in urban areas had longer birth intervals than their rural counterparts (*p* < 0.05) [18, 24]. Another study from Tanzania revealed that short birth spacing was higher among women that resided in rural areas than their urban counterparts (50% versus 45%), and the difference was statistically significant (*p* < 0.001) [15]. Similar cross-sectional study from Ethiopia showed similar conclusion [17].

Economic status of respondents was also found to be a strong predictor of birth interval. Study done in Saudi Arabia indicated that shorter birth intervals were independently predicted by lower family income [13].

In contrast, study conducted in Lemo District, Ethiopia, showed that the median length of birth interval grew as one shift from lowest quartile to highest quartile of the wealth index (AOR = 0.49, 95% CI (0.25, 0.96)) [17]. Evidence from Ethiopian DHS also indicated that there is no statistically significant difference in the median birth interval by wealth quintiles, although births to women in the highest wealth quintile have the longest median birth interval (38.9 months) [5].

#### 3.2.2. Birth History of Respondents

With respect to place of delivery, study in Rufiji, Tanzania, showed that the interbirth intervals for which the index child was born elsewhere other than in a health facility were more likely to be less than the recommended minimum length compared with those for which the index child was born in a health facility (OR = 1.81, 95% CI (1.68–1.94)) [15].

The same study from Tanzania indicated that interbirth intervals corresponding to twins or multiple births of the index child were less likely to be shortly spaced compared with those corresponding to singleton birth of the index child (OR = 0.76, 95% CI (0.59–0.97)) [15].

According to study done in Manipur, the median birth interval of respondents was indicated to decrease with the increasing in the age at first marriage of women (*p* < 0.05) [11]. Other studies done in Jordan and Ahvaz (Iran) indicated similar conclusion [21, 25].

Study in Nepal also indicated that age when they have started sexual relationship with their spouses has statistically significant positive association with birth interval [18]. Study in Tanzania also indicated that age at first delivery has statistical association with birth interval [24].

Study in Pakistan indicated that preceding birth interval has statistical association with current birth interval [22]. Sex of the index child was found to be a strong determinant of birth interval among respondents. Study from Manipur showed that average birth interval was significantly shorter for women with a preceding birth of a female child (*p* < 0.01) [11]. Study from Saudi Arabia, Babol, Jordan, and Tanzania provided similar evidence [13, 14, 21, 24]. According to study done in Oromia Region, Ethiopia, women who had female index child were more likely to have short birth interval compared to mothers who had male index child [23]. However, evidence from EDHS 2011 showed that sex of the index child has no statistically significant association with birth interval [5].

Study in Babol, Northern Iran, indicated that respondents with history of still births and child mortality showed statistically strong association with birth spacing [14]. Another study done in Manipur indicated that mothers who had previous infant mortality were more likely to have long birth intervals as compared to their counterparts (*p* < 0.01) [11].

Survival status of the preceding child was also found to be a major predictor of birth intervals. According to the study done in Pakistan, mothers whose previous child was dead were more likely to have short birth interval compared to those with surviving child [22]. Report from EDHS 2011 indicated similar conclusion [5].

Regarding pregnancy plan, study done in Denmark indicated respondents with planned pregnancy were more likely to have longer birth intervals than those who reported that their pregnancy occurred unintentionally [20]. The health of the mother, the wellbeing of the last child, and the family's financial situation were also stated as influencing the decision of women for spacing the next birth [26].

Parity has also significant positive impact on the risk of having subsequent birth. Study in Manipur and Babol, Northern Iran, indicated that birth interval increases with increasing parity (*p* < 0.05) [11, 14]. Another study in Denmark showed that irregular menstruation showed a significant correlation with birth interval [20]. However, evidence from EDHS 2011 indicated that birth order has no effect on birth interval [5].

#### 3.2.3. Breast-Feeding Practice

According to the study conducted in Iran among multiparous women, duration of breast-feeding was found to be an independent predictor of birth intervals. It showed that women who breast feed their child for longer than 24 months were more likely to have longer birth intervals than those who feed for less than 6 months (AOR = 0.01, 95% CI (0.004–0.046)) [14]. Other studies from Manipur, Jordan, Pakistan, Ahvaz (Iran), Egypt, and Nigeria reported similar conclusions [11, 21, 22, 25, 27, 28].

As opposed to the above, finding from Lemo District, Ethiopia, showed that duration of breast-feeding has inverse relationship with birth intervals. Women who breast feed for 7 to 12, 13 to 23, and 24 and above months were found to be significant predictors of short birth interval [17].

#### 3.2.4. Knowledge and Attitude of Women about Contraception and Birth Spacing

Contraceptive method utilization was also cited as one of the major determinants of length of birth intervals. Study done in Manipur indicated that women who used modern contraception were more likely to have longer birth intervals than those who never used any contraceptive method (*p* < 0.01) [11]. Similarly, study from Jordan, Ahvaz (Iran), and Egypt indicated that use of modern contraceptives showed a significant positive correlation with birth interval [21, 25, 27]. Other studies from Ethiopia also suggested similar evidence [17, 23].

Regarding Knowledge of respondents, optimal birth spacing until the next pregnancy for Egyptian women is generally understood to refer to a 2-3 year period [26].

According to study done on Saudi Arabian women, 90.8% of the respondents had used one method for birth spacing, the most popular being oral contraceptive pills (65.1%) followed by the intrauterine device (24.5%) and breastfeeding (20.6%) [19].

About half of the women in this study lacked awareness about the known benefits of longer birth intervals and adequate child spacing. A significant proportion of the respondents were not aware that longer birth interval could lead to improvement in the child's height (60.1%), weight (45.8%), intelligence (50.7%), and school performance (41.5%) as well as lowering the risk of infant and perinatal mortality and morbidity (47.3%). The majority (88.5%), however, perceived that long birth intervals would decrease the risk of maternal mortality and morbidity [19].

According to the study in Saudi Arabia in which women were asked about their ideal birth interval, 12 (2.8%) women stated that they had no preference, 22 (5.2%) preferred <2 years, 123 (28.2%) preferred 2 years, 159 (36.5%) preferred 3 years, and 120 (27.5%) preferred >3 years. Test for preferred and actual birth interval showed statistically significant relationship (*p* < 0.001) [19]. In this study, the reasons given for preferring a short birth interval (<2 years) included husband's wish (50.0%), ease of taking care of children in quick succession (31.8%), desire to complete family quickly (31.8%), and dictation of religion (18.2%), whereas the reasons given for preferring a longer birth interval included good physical growth of children (38.7%), good health of children (43.0%), and better maternal health (58.1%) [19]. Another study in Jordan and Ahvaz (Iran) showed that ideal birth spacing has strong positive association with birth interval [21, 25].

Different studies indicated that the majority of mothers had positive attitude towards birth spacing [15, 19, 29, 30]. According to study in Jordan, the majority of husbands had positive attitude towards longer birth spacing and statistically associated with education and monthly income [31]. Health facility based cross-sectional study in Saudi Arabia showed that husbands' encouragement of interbirth spacing was a significant predictor of longer interbirth interval. It indicated that mothers whose husbands encouraged them to space their births are more likely to have longer birth intervals than their counterparts [13].

In summary, evidence from different literatures revealed that age of the mother, occupational status of the mother, educational status of the mother, place of residence, monthly income, husband's educational status, husband's occupational status, sex of the index child, survival status of the index child, parity, duration of breast-feeding, and modern contraception use were factors found to be independent predictors of interbirth intervals.

## 4. Objectives

### 4.1. General Objective

The general objective was to assess determinants of interbirth interval among child-bearing age women who have at least two consecutive live births in Arba Minch Zuria Woreda, SNNP, Ethiopia, 2014.

### 4.2. Specific Objective

- The specific objective was to identify determinants of interbirth intervals among child-bearing age women having at least two consecutive live births.

## 5. Hypothesis Testing

*Null Hypothesis*. Determinants of interbirth intervals are the same among cases and controls.

## 6. Methods and Materials

### 6.1. Study Area

This study was conducted from January to July 2014 in Arba Minch Zuria District, demographic and health survey joint venture project (research) site. In this district, demographic and health survey project was launched by Ethiopian public health association and center for disease control of USA (EPHA and CDC) in collaboration with Arba Minch University in eight rural and one suburban villages having three climatic zones (highland, semihighland, and lowland). It has been an input for local, regional, and national health care planning, policy making, and research projects by generating dependable data related to birth, death, and migration events.

Arba Minch Zuria is one of the districts found in south nations, nationalities, and people's regional state. It is located in the Great Rift Valley. The administrative town of this district, Arba Minch, is located about 502 Kms to south west of Addis Ababa, the capital city of Ethiopia. The district is situated 1285 meters above sea level. Gamo as ethnicity (69.53%) and Protestants (53.91%) as religion are the dominant ones. According to the report from central statistical agency, 2007, this district has a total population of 164,529, of which about 82,199 were men and 82,330 were women [32]. Regarding health care facility, the district has only three health centers operating currently. It has no hospital of any level. The subdistricts have difficult topography and limited road infrastructures and most of them are reached on foot.

### 6.2. Study Design and Period

Community based unmatched case control study design supplemented by qualitative method was employed to assess determinants of interbirth interval among women of child-bearing age who had at least two consecutive live births in Arba Minch Zuria Woreda research site from February to April 2014.

### 6.3. Source and Study Population

#### 6.3.1. Source Population

Source population was all child-bearing age women found in Woreda who experienced at least two successive deliveries and the last delivery occurred within the past five years prior to the data collection.

#### 6.3.2. Study Population

Study population was randomly selected women of child-bearing age living in Woreda who have at least two consecutive births, the last delivery being within the past five years prior to the survey on which the actual study was to be conducted.

*Cases*. They were randomly selected women of child-bearing age with at least two consecutive deliveries who have history of short birth intervals (birth interval of less than 3 years between two successive births).

*Controls*. They were randomly selected women of child-bearing age who had at least two consecutive births and had a history of optimum birth intervals (birth interval of 3–5 years between two successive births including 3 and 5 years).

### 6.4. Inclusion and Exclusion Criteria

Inclusion criteria are as follows:

- For controls: women who gave live birth within the last 5 years and have at least two consecutive live births with birth interval of 3–5 years between the latest two successive live births inclusive.
- For cases: women who gave live birth within the last 5 years and have at least two consecutive live births with birth interval of less than 3 years between the latest two successive live births.

Exclusion criteria are as follows:

- Clients who were mentally ill and could not make logical judgment.

### 6.5. Sample Size Determination

The sample size was determined by the formula used for unmatched case control study using Open EPI INFO version 3.5.1 software by taking account of the major determinant factors.

Accordingly, a minimum detectable OR (Odds Ratio) of 2, a 5% level of precision, a power of 90%, and a 2-to-one allocation ratio of optimum birth interval (controls) to short birth interval (cases) were assumed.

An additional nonresponse rate of 10% was also considered. The proportion of mothers who utilized modern contraception among controls was also considered to be 68.4% [23]. Based on the above assumptions, the final sample size was determined to be 636 (212 cases and 424 controls). Contraception utilization rate was chosen as an independent variable since it gave a maximum sample size.

For the qualitative method, focus group discussions on purposely selected cases and controls as well as their husbands were conducted using open ended questions until information is saturated. In the study, sixteen FGDs were conducted involving a total of 128 participants (64 males and 64 females). Eight groups were from parents who had interbirth interval less than 3 years (four groups of husbands and four groups of mothers) and eight groups were from parents who had after birth an interval of three to five years (four groups from each of husbands and mothers).

### 6.6. Sampling Procedure

**Figure 1:**
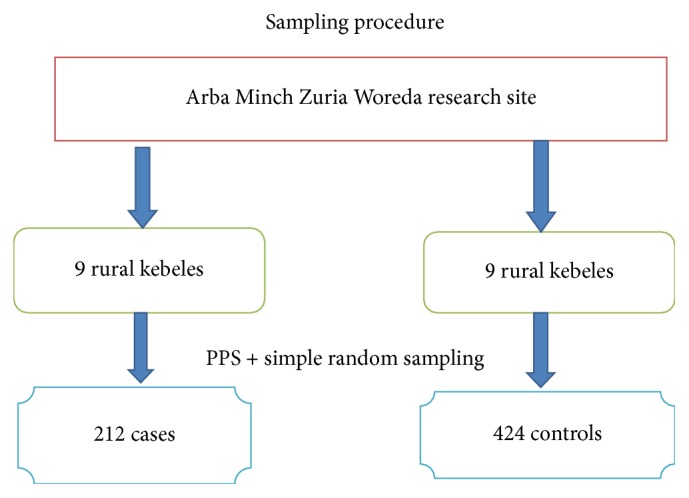
Schematic presentation of sampling procedure for Quantitative study among child-bearing age group mothers in Arba Minch Zuria Woreda, SNNPs, Ethiopia, 2014.

Regarding the sampling procedure, first, all the nine rural kebeles found in Arba Minch Zuria District DHS research site were selected (Figure 1). Then, house to house visit (census) was conducted in all the kebeles to identify women who fulfill the inclusion criteria (cases and controls) by having registered the birth date of the last two children of a family with their corresponding household identification number. To determine children's birth dates, immunization cards were used. For those who were not immunized, community based health extension workers were consulted. Using respective household identification number, frames of households containing study subjects defined as cases and controls were prepared for each kebele. Then, probability proportional to size allocation technique was employed to determine the study participants from each kebele as well as cases and controls. Finally, child-bearing age women who had at least two consecutive live births and whose last delivery was within the past five years prior to the survey were selected using simple random sampling technique from the existing sampling frame. Whenever more than one eligible respondent was found in the same selected household, only one respondent was chosen by lottery method. For participants who were not present at the time of data collection, at least three visits were made to trace them. Whenever more than one eligible respondent is found in the same selected household, only one respondent will be chosen by lottery method. If the participant in the selected household is not present at the time of data collection, at least three revisits will be made to interview the woman.

*Participant Selection for Qualitative Study.* Study participants were selected purposively in the manner that they can be representative of the mothers with different reproductive history and residence. All the nine rural kebeles found in Arba Minch Zuria District DHS research site were first considered. Then, four kebeles (two from the highland and two from the lowland areas) were selected. The highland and lowland areas have different socioeconomic status and therefore mothers and their husbands were chosen from both to adequately represent the study area.

Once the study kebeles were identified, parents (mothers and their husbands) living in the four kebeles and having different birth histories were selected purposely from the list of households found in each kebele. Individuals who were believed to be the most informative about the subject under study were selected. Being married, having not been involved in any previous survey, and being not related with each other were additional criteria used to select the study participants. To assist in finding and recruiting the discussants, community based health extension workers, development army leaders, and kebele administrators were consulted. Once the women were chosen, their husbands were also recruited to the study in order to gain a full understanding of the knowledge and perceptions of birth interval. Mothers and their husbands who were sick and incapable of making discussions at the time of the study were not included. Discussants were classified by sex, area of residence, and level of birth interval to capture heterogeneity among different subgroups and homogeneity within a group.

To determine children's birth dates and their respective birth intervals, immunization cards were referred. For participants whose children were not immunized, community based health extension workers were consulted since they have full and up-to-date document of all vital statistics. Finally, child-bearing age women who gave birth with in the last five years and had at least two consecutive live births were invited to participate in the FGDs.

### 6.7. Data Collection Tools and Procedures

This study obtained data from the interviews of mothers residing in sampled households. Structured and pretested questionnaire was employed to obtain information on obstetric history, socioeconomic status, contraception use, breast-feeding practice, and attitude towards birth spacing and family planning. The questionnaire was adopted from different previously done studies and adapted to the local context of the study area [12–14]. It was prepared first in English and then translated to Amharic by the language expert for the data collection purpose. To check its consistency, the questionnaire was translated back to English by another language expert.

Eight high school graduates who were familiar with the local language and customs were recruited as interviewers. Four B.S. degree holding health care workers supervised the data collection process. Data collectors and supervisors were trained for three days on census procedures, questions included in the questionnaire, interview techniques, and importance of privacy and confidentiality of the information obtained from the respondents. Before conducting the main study, pretest was carried out on 5% of the sample size (20 cases and 20 controls) from one kebele, which was not included in the main study. Based on the result, data collectors were reoriented and the questionnaire was modified as appropriate. Data collected from each respondent were checked for completeness, clarity, and consistency by the principal investigator and the supervisors at the end of each data collection day.

For the qualitative study, data were collected through focus group discussions (FGDs) with mothers and their husbands to explore their perceptions about interbirth intervals. Open ended semistructured and flexible questionnaire was used as a guide for the discussion process. Separate FGDs for men and women were held. The discussions were conducted in a quiet room according to the preference of discussants to enable them to speak more freely about their perceptions. Two moderators and one field assistant were recruited to collect the desired information. At the begging of the discussion, the moderator introduced all participants, explained the topic and purpose of the discussion, and made them aware that each and every opinion was important and wanted and that they should feel free to express it.

Husbands' FGDs were moderated by the principal investigator. Since mothers could relatively openly express their ideas with females without being afraid and shy, FGDs for mothers were moderated by registered female B.S. degree holding nurse. Data were tape-recorded by a research assistant after thorough communication had been made with discussants. Each FGD lasted within 90–120 minutes. The number of FGDs was determined by the saturation of ideas. Discussion questions included perceived knowledge about short and optimal birth intervals, perceived disadvantages of short birth intervals, perceived advantages of optimal birth intervals, reasons for having short birth intervals, and decision-making about interbirth intervals.

### 6.8. Study Variables

Independent variables are as follows:

- Sociodemographic factors: occupational status of the mother, marital status of the mother, ethnicity, educational status of the mother, religion of the mother, husband's educational status, and husband's occupational status.
- Economic status: wealth index.
- Birth history: age at first marriage, age at last delivery, number of living children, sex of the preceding child, delivery place of the preceding child, survival status of the preceding child, pregnancy plan, multiple pregnancy, ANC visit during previous pregnancy, women's decision-making power, and husbands belief regarding birth spacing.
- Breast-feeding practice, knowledge on birth interval, attitude towards birth spacing and family planning, and knowledge and practice of modern contraceptive methods.

Dependent variables are as follows:

- Short interbirth interval.

### 6.9. Operational Definitions

Some operational definitions are given as follows:

- Maternal death: deaths of women while pregnant or within 42 days after termination of pregnancy, irrespective of the duration and site of the pregnancy, from any cause related to or aggravated by the pregnancy or its management, but not from accidental or incidental cause [4].
- Positive attitude: mothers were considered as having positive attitude towards optimal birth spacing and contraception if they scored above mean correct answers from attitude measuring questions.
- Negative attitude: mothers were considered as having negative attitude towards optimal birth spacing and contraception if they scored mean and below correct answers from attitude measuring questions.
- Short interbirth interval: it refers to less than 3 years' birth interval between the birth of the child under study and the immediately preceding live and surviving birth to the mother [6].
- Optimal birth interval: it denotes to 3–5 years' birth interval (including 3 and 5 years) between the birth of the child under study and the immediately preceding live and surviving birth to the mother [6].
- Permanent resident: mothers who have been living in Arba Minch Zuria Woreda for more than six months.

*Estimation of Household Wealth Index.* Household wealth status was estimated by principal component analysis based on fourteen household variables (source of drinking water, presence of own farmland, size of own farm land, type of toilet facility, electricity, radio, mobile phone, roof of house with corrugated iron sheet, sleeping bed, and number of cows, oxen, horses/mules/donkeys, goats/sheep, and hens). SPSS version 20 software was used to perform principal component analysis (PCA). Both Kaiser-Meyer-Oklin (KMO) and Bartlett tests were checked. Finally, wealth status was categorized into five groups and ranked from poorest to wealthiest quintile.

*Outcome and Exposure Measures.* The outcome variable was the birth interval defined as the period from the date of birth of the previous child to the date of birth of the last child. The birth intervals between two consecutive live births were analyzed for women aged 15–49 years who reported that they had at least two deliveries and the last delivery occurred within the past five years prior to the data collection. Dummy variables were created for this variable: birth interval less than 3 years (short birth interval) and birth interval 3–5 years inclusively (optimal birth interval). Short birth interval was coded as “1” and optimal birth interval was coded as “0.”

### 6.10. Data Quality Assurance

Data quality was ensured during collection, coding, entry, and analysis. During data collection, adequate training and follow-up were provided to all data collectors and supervisors. The principal investigator and supervisor conducted a day-to-day on-site supervision during the whole period of data collection.

At the end of each day, the questionnaires were reviewed and checked for completeness, accuracy, and consistency by the supervisor and investigator and corrective discussion was undertaken with all the research team members. Remarks were given during morning times on how to eliminate or minimize errors and take corrective actions timely. Codes were given to the questionnaires during the data collection so that any identified errors are traced back using the codes.

Data were first checked manually for completeness and then it was coded and entered into Epi-Info version 3.5.1 statistical software and cleaned thoroughly before being transferred to SPSS version 16 for further analysis. The data were further cleaned by visualizing and calculating frequencies and sorting in SPSS. Corrections were made according to the original data.

### 6.11. Data Analysis

After appropriate coding, the data were entered using Epi Info version 3.5.3 and exported to SPSS version 20 software for analysis. Univariate analysis was computed for each independent variable to assess their individual proportion. Then, bivariate analysis was executed to examine crude association of predictors with short interbirth intervals. Finally, variables which had *p* value less than 0.3 on bivariate analysis were selected as candidates for multivariable analysis. In multivariable logistic regression analysis, the independent effect of predictors on short interbirth interval was examined. Backward stepwise LR was used to identify variables which had the largest contribution to the model. Odds Ratio and 95% CI were used to measure the statistical association. *p* value 0.05 was used to determine the statistical significance of the tests. Finally, the results were presented in texts, tables, and graphs.

For the qualitative data, all the audiotaped FGDs were transcribed verbatim after listening again and again. All observations made by the field assistant were recorded as field notes. Then, translation was made from Amharic (official and working language) to English. To assure the validity of the translation, another person, proficient in both languages, checked and commented on it. Both transcription and translation of FGDs were made by the investigators. When there was no consensus during translation, both the principal and the coinvestigators replayed the original recordings and compared them with the transcripts until agreement was reached. Finally, the principal investigator double-checked against the original recordings and the research team analyzed using content analysis.

Prior to coding, reading and rereading were done by the principal investigator. Summarizing of the word-for-word transcripts was carried out. The transcripts (questions and their respective responses) were prepared in Microsoft Word, saved in plain text, and entered to open code software (qualitative data analysis software). Once the transcript is entered, the investigators read the transcript again in order to become familiar with the responses. Then, the transcripts were coded according to the emergent categories. The principal investigator conducted the coding process. After completion of the coding process, themes were developed and classified. Contents were also supplemented with illustrations of direct quotes from the discussion. Then, final themes were compared among various groups' discussants to know the differences and similarities according to their perspectives about factors influencing short birth intervals. The findings were finally presented in the form of narratives.

### 6.12. Ethical Consideration

After approval, ethical clearance was obtained from Institutional Review Board (IRB) of College of Medicine and Health sciences, Arba Minch University. Then, official letter was written from College of Medicine and Health Sciences to Arba Minch Zuria District health office. Permission letters from district health office were processed before starting data collection. At the beginning of the data collection, informed consent was obtained from each respondent after thorough explanation of the purpose and the procedures of the study. For respondents whose age is less than 18 years old, informed consent was planned to be obtained from their parents/guardians. However, since minimum age of the respondents was 19 years, written informed consent was secured only from the respondents themselves. Mothers were also informed that all the data obtained from them would be kept confidential and anonymous. To ensure confidentiality, names of respondents were replaced by code numbers.

### 6.13. Dissemination Plan

The findings of this study will be communicated to College of Medicine and Health Sciences, Arba Minch University, SNNP Health Bureau and Arba Minch Zuria Woreda Health Office. The findings will also be presented in various seminars/workshops and publication will be considered in scientifically reputable journals.

## 7. Result

### 7.1. Quantitative

#### 7.1.1. Socioeconomic Characteristics of Participants

Six hundred thirty-six child-bearing age women who had at least two live births were interviewed making a response rate of 100%. The mean age of the respondents was 31 years (SD ±5.16). Eighty-one (38.1%) of the cases and 181 (42.7%) of the controls were within the age range of 25–29 years. The majority (107) (50.5%) of the cases and 226 (53.3) of the controls were married at the age of 18 years or less. Regarding educational status, one hundred sixty-nine (79.7%) of the cases and 203 (48.0%) controls had not attended formal education. The poorest wealth index was computed at 63 (29.7%) and 64 (15.1%) in the cases and controls, respectively (Table 1).

Among the above socioeconomic factors, ethnicity, educational status of both parents, and wealth index of the respondents showed crude statistical association with short interbirth interval. The odds of experiencing short interbirth interval were higher for mothers who were illiterate or unable to read and write as compared to those who attended formal education. Similarly, as wealth index of respondents decreased, the likelihood of having short interbirth interval was high (Table 1).

#### 7.1.2. Obstetrics, Breast-Feeding, and Contraception History of Mothers

Ninety-nine (46.7%) of the cases and 216 (50.9%) controls were reported to have three up to four live children. Five (2.3%) of the cases and 11 (2.6%) of the controls had still birth between the last two live births. Ninety-nine (46.7%) and 299 (70.5%) of cases and controls, respectively, had planned pregnancy. Twenty-two (10.4%) of the cases and 57 (13.4%) of the controls gave birth in health institution for their preceding child. The majority (178) (84.0%) of the cases breast fed for less than 24 months, while 383 (90.3%) of the controls breast-fed for 24 months or above. Sixty-six (31.1%) of the cases and 283 (66.7%) of the controls utilized modern contraceptive method after the delivery of the preceding child but before they got pregnant with the last child (Table 2).

On bivariate analysis, sex of the preceding child, pregnancy plan for the last child, antenatal care follow-up during the preceding pregnancy, contraceptive utilization before getting pregnant for the last child, and duration of breast-feeding for the preceding child had showed significant statistical association with short birth interval. Female sex of the preceding child was positively associated with experiencing short birth interval. Similarly, the odds of experiencing short birth interval were about 3 times higher for mothers who did not have pregnancy plan for their last child than those who had a plan to get pregnant. The likelihood of having short birth interval was 3 times higher for mothers who did not attend ANC follow-up during the preceding pregnancy as compared to those who attended it. Mothers who did not use modern contraceptive method before getting pregnant with the last child were 4 times more likely to experience short birth interval as compared to those who used it. Duration of breast-feeding of the preceding child had also showed significant statistical association. The odds of having short birth interval were about 49 times higher for mothers who breast-fed their preceding child for less than 24 months as compared to their counterparts who breast-fed for 24 months or more (Table 2).

#### 7.1.3. Multivariable Analysis of Factors Affecting Short Interbirth Interval

To determine the factors independently affecting interbirth interval of the last two children born to the study participants, bivariate and multivariate analysis was carried out. In multivariable logistic regression analysis, educational status of the mother, contraceptive utilization, duration of breast-feeding, sex of the preceding child, age during delivery of the last child, and wealth index were found to be independent predictors of short birth interval (Table 3).

Level of education showed strong statistical association with short interbirth interval. Mothers with no formal educational were about 3 times (AOR = 3.40, 95% CI: (**1.80**–**6.43**)) more likely to have short interbirth interval as compared to those who attended formal education. The other strong predictor of interbirth interval was utilization of contraceptive methods. The odds of having short birth interval were higher among mothers who did not use modern contraceptive method before getting pregnant with the last child (AOR: 3.01, 95% CI: (**1.68**–**5.39**)) than those who used this method.

Similarly, mothers who breast-fed the preceding child for less than 24 months were more likely to have short interbirth interval than their counterparts of mothers who breast-fed for 24 months or more (AOR: 60.19, 95% CI (**31.61**–**114.59**)). Sex of the preceding child has also revealed a significant association with interbirth interval. Mothers whose preceding birth was female were about 7 times (AOR: 6.79, 95% CI (**3.65**–**12.63**)) more likely to experience short birth interval than those whose child was male. Wealth index of the mother was also a strong predictor of short birth interval. The odds of having short interbirth interval were higher for mothers who belong to the poorest wealth index than the richest ones (AOR: 14.33, 95% CI (**4.65**–**44.15**)).

### 7.2. Qualitative Result

In this study, sixteen focus group discussions involving a total of 128 participants (64 males and 64 females) were conducted. Eight groups were from parents who had interbirth interval less than 3 years (four groups of husbands and four groups of mothers) and eight groups were from parents who had interbirth interval of three to five years (four groups of husbands and four groups of mothers).

All discussants were health development army members. Short and optimal birth spacing participants ranged in age from 22 to 35 years and from 23 to 44 years, respectively. Educationally, the majority of the mothers and husbands did not attend formal education. Almost all of the women and men discussants were housewives and farmers, respectively. Their parity and interbirth intervals ranged from three to nine and one to five years, respectively. The contents of the discussion included perceived knowledge of women and men about short and optimal birth intervals, perceived disadvantages of short birth intervals, perceived advantages of optimal birth intervals, reasons for having short birth intervals, decision-making about interbirth intervals, and the effect of religion on decision-making about interbirth intervals.

#### 7.2.1. Definition of Interbirth Spacing

Almost all mother and husband discussants defined birth spacing correctly. For all of them, birth spacing until the next delivery was understood as extending the period of time between the two consecutive births in the manner that could allow the mother to recover from pregnancy, labor, childbirth, and lactation, replace her nutritional stores, and provide time for the last child to self-feed and take independent care. The duration of time for short interbirth interval as defined by majority of the women and men discussants conformed to the world health organization recommendation. According to them, short birth interval refers to birth interval of less than three years. However, discussants were unable to differentiate between optimal and long birth intervals. The majority of the discussants who were experiencing short and optimal birth intervals realized that birth interval is said to be optimal when it is greater than three years.

#### 7.2.2. Perceived Advantage and Disadvantage of Short Interbirth Intervals

*Perceived Advantages of Short Birth Spacing*. Irrespective of their birth spacing status, the majority of the discussants from all categories underlined that short interbirth intervals are by no means important for the health of the mother, the new born baby, the father, the rest of the children at home, and the overall quality of life of the family. They reported that it deteriorates the health and economic status of the family. They further justified that mothers were having short birth intervals not because they believed it is beneficial but because of the influence of their husbands and religion, lack of health care information, and inaccessibility of reproductive health care services.

*…from my experience women believe that closely spaced births are beneficial to neither of the family members. They are giving birth with in short time intervals because of different negatively influencing internal and external factors. (A 29-year-old mother from short birth spacing group).*

Very few advantages were noted by some of the discussants. Some women and husbands who were experiencing short birth intervals reported that having closely spaced births was advantageous to early complete child birth before the woman gets weak: “*giving birth when the mother becomes old exposes to economic and health problems. Moreover, when the mother becomes sick as a result of labor and delivery she does not produce enough milk to the child” (a 34-year-old mother from short birth spacing group).* Some also believed that if the index child was female the woman should frequently give birth until she gets a boy.

*Perceived Disadvantages of Short Interbirth Spacing*. The majority of the discussants noted birth interval as a period for parents and children to make adequate preparations to receive a new born child and give appropriate care. It is the time when the mother primarily gets ready physically, mentally, and economically. Unless this interval is planned and sufficient preparation is made, the outcome will be devastating to the health of the mother, the fetus in the womb, the father, and the rest of the children at home:* “those who have closely spaced births are nutritionally deprived and not healthy. Besides, it decreases the productivity of the family, particularly the mother” (a 30-year-old husband from short birth spacing group)*. According to the discussants, in most cases the mother is the primary care giver. Therefore, her health condition has a direct impact on the children in particular and the family as a whole. They noted that childbirth and lactation weaken the mothers' physical condition. Any childbearing related strain before the completion of at least 3 years would endanger her life, lead to the lactating period being reduced and thereby directly affecting the child's nutrition and growth. In addition to this, physical weakness means that the capacity of the woman to execute household responsibilities is reduced:* “yeah, if the mother got pregnant before she recovers, she will not be healthy and never be productive to the family. When the mother is sick, the infant will be sick too” (a 30-year-old husband from short birth spacing group)*. This could be a big problem particularly in communities whose socioeconomic status is poor. Thus, the disadvantages of short birth spacing were classified into three subthemes: physical, psychological, and financial disadvantages.

*Disadvantages for the Mother.* There was a general agreement among all focus groups that optimally spaced births allow the mother to recover from pregnancy, child birth, and lactation, replenish her nutritional stores and provide time for the last born to self-feed and care. On the other hand, if child birth is closely spaced the mother faces multiple problems. Mothers' health condition would be deteriorated through bleeding, anemia, nutritional deficiency, and child birth related trauma. Psychologically, it was felt that the mother would experience higher stress and exhaustion, be less relaxed, and not have adequate time to take care of herself, her last born child, her home, and her husband.

*“If the mother does not get sufficient rest after the preceding birth when she can regain important nutrients, and recovers from physical and psychological health problems the health of the mother herself, and her new born child will be endangered. Yeah, the family will not be stable and peaceful because the disturbance of health condition.” (A 35-year-old woman from optimal spacing group)*. Unhealthy and psychologically unstable mother would be more likely to have a negative effect on the wellbeing of the newborn.

*Disadvantages for the Husband.* Almost all discussants understand that closely spaced birth intervals negatively affect the father in terms of health and economic status of his family. They noted that when the mother is sick and has many closely spaced children demanding beyond the household capacity, the father would psychologically be ill:* “if the number of children is increasing, demand for additional resources and further poverty will be the end result. It also keeps children out of school. These all are burden to the father and make him psychologically sick” (a 32-year-old husband from optimal spacing group)*. He will not be productive in his work and will lead a stressful life. Financially, he might not have an opportunity to save money and invest in his family and would be overwhelmed by the needs of his growing family. Some discussants indicated that if the husband did not receive the attention and comforts he expected, he might consider taking another wife to provide the care and comfort missing from the relationship with his current partner. This seriously affects the life of the family as a whole.

*Disadvantages for the Preceding and the New Born Child.* Participants believed that mothers who space their children have more time to assure their children are clean, well-fed, and loved, have adequate clothing and affection, and are taken care of when sick. They felt these advantages would ensure that the child grows up healthy and cared for, with greater physical, mental, and emotional wellbeing. On the other hand, if children are closely spaced, the last two children can partly or wholly lack all the above activities mentioned. If both of the children are dependent on care as a result of close spacing, the attention of the mother and the resources will be divided between them. They do not get adequate clothing, are not appropriately feed or cleaned, and do not share the love of the mother:* “if children are born in less than 3 years, they become short and thin. They remain seated. They never walk on time. Besides the health of the mother and father will be affected” (a 36-year-old husband from optimal spacing group)*. In addition, the duration of breast-feeding will be short and the preceding child will never get the entire nutrient he has to from his mother: “*if children are born in less than 2 years they become poorly nourished because of inadequate breast-feeding duration” (a 28-year-old mother from optimal spacing group).* Discussants also believed that the potential for jealousy between young siblings could also be another problem, as the last born would compete with the newborn for his/her mother's attention and resources.

*Decision-Making Process about Birth Spacing.* There was no real consensus as to who makes the ultimate decisions regarding spacing. Almost all of the mothers and their husbands who were conforming to the recommended range of birth spacing (optimal birth interval) reported that decision about when to have the next birth is shared between the couples (husband and wife). Regardless of who has the upper hand in decision-making, it was reported that planning and discussion accompany the final decision. Even if opinions differ, each partner has to persuade the other when pregnancy should not occur. On the other hand, the majority of discussants who had short birth intervals indicated that the decision-making power is vested in the hands of the husband. The majority of husbands and mothers believed that the husband had the decision-making role regarding the timing of subsequent pregnancies. Based on his financial position within the family, he had the final say. Discussants added that husbands view their decision as the final word and the wives' role as trying to persuade him is unlikely. Thus, decision-making is skewed in favor of the man. Some optimal birth spacing mothers noted that when the mother lives in an extended family, the decision-making power is mainly vested in the mother. It is the mother or sometimes the father who had the final say as when the woman should get pregnant.

*Reasons for Choosing Short Birth Intervals.* Discussants from short birth spacing groups indicated that community and health care service factors deter women from adhering to optimal birth spacing. Lack of information about the benefit of contraceptive methods and optimal birth spacing was noted as the major obstacle by majority of the discussants from both categories:* “the major reason why women are experiencing short birth interval is lack of information about family planning methods and birth spacing” (a 34-year-old husband from short spacing group)*. Moreover, inaccessibility of the reproductive health care service was repeatedly underlined by short spacing mothers and their husbands from the high land areas. Shortage of public transportation service and road infrastructures compounded by the difficult topography of the district makes family planning utilization impossible in the area. The other factors preventing women from having optimal birth spacing are reliance on “clean lactation” and the negative side effects of contraceptive use, which tend to make women partially or completely stop using them and experience method failure. Just the fear of side effects deters some women from using contraceptives:* “as the contraceptive method I was using is not confortable [sic] for me I stopped using it and give birth at a short period of time” (a 30-year-old husband from short spacing group)*. Some of the discussants from short spacing group mentioned that husband, parent, and religious influence could also prevent women from using contraception and practicing optimal birth spacing:* “fear of being divorced makes women experience short birth spacing. Lack of education prevents me from influencing my husband and my life” (a 29-year-old mother from short spacing group).* Few discussants mentioned that if the preceding child is a female they have an intention to give birth frequently until they get a boy.

## 8. Discussion

Understanding the determinants of short interbirth interval is critical for many Sub-Saharan African countries like Ethiopia, where perinatal mortality and fertility remain alarmingly high. Thus, this community based case control study identified factors influencing short interbirth interval among mothers who had at least two live births with their last child born within the last five years in Arba Minch Zuria District, Ethiopia.

Even though no statistical association was revealed, the majority of the respondents defined optimal birth spacing correctly. Similarly, almost all FGD male and female discussants, irrespective of their level of birth interval know the year limits for optimal birth spacing. However, discussants were unable to differentiate between optimal and long birth intervals.

In this study, the odds of having short interbirth interval were higher among mothers who had no formal education as compared to their women counterparts who attended formal education. This finding is consistent with evidences from study conducted in Saudi Arabia, Nepal, Jordan, and Pakistan [13, 18, 21, 22]. This can be partly explained by the fact that educated women are well informed about optimal health care choices and have greater autonomy to make decisions and use quality health care services. In line with evidences from studies done in Manipur, Ethiopia, Jordan, Ahvaz (Iran), and Egypt [11, 17, 21, 25, 27], the finding of the present study indicated that mothers who did not utilize modern contraceptive method before they got pregnant with their last child were more likely to experience short interbirth interval than those who used one. Sex of the preceding child has also revealed a significant association with birth interval. The finding of the present study revealed that the odds of having short interbirth interval increased for mothers whose preceding birth was female than mothers who had males. Study findings from Manipur, Saudi Arabia, Babol, Jordan, and Tanzania provided similar evidence [11, 13, 14, 21, 24]. This may be attributed to the reason that a son is considered as a potential economic asset to the family as a whole and it is therefore less likely for mothers to exercise long time breast-feeding or utilize modern contraceptive method as a means of birth control until they get the desired number of sons.

Similarly, mothers who breast-fed the preceding child for less than 24 months were more likely to have short interbirth interval than their counter parts of mothers who breast-fed for 24 months and above. This study finding was congruent with evidences from Manipur, Iran, Jordan, Pakistan, Ahvaz (Iran), Egypt, and Nigeria [11, 14, 21, 22, 25, 27, 28]. This may be due to the fact that breast-feeding extends period of interbirth interval through negative hormonal feedback.

Wealth index of the mother was also a strong predictor of short interbirth interval. Consistent with evidence from study done in Saudi Arabia [13], the odds of having short interbirth interval were higher for mothers who belonged to the poorest wealth index than the richest group of mothers. In contrast, study conducted in Lemo District, Ethiopia, showed that the length of interbirth interval increased with increasing wealth index [17]. This can be partly explained by the fact that wealthy women are more likely to access health care information and afford health care services and materials and thus can easily apply scientifically recommended interbirth spacing.

Statistically significant association was also seen between short birth interval and age of the mother. In line with study from northern Iran, Tanzania, Saudi Arabia, Nepal, Denmark, Jordan, and Pakistan [14–16, 18, 20–22], this study revealed that mothers who belonged to the age range of 30–34 years were more likely to have short birth interval as compared to those whose age was 35 years and above. Evidence from EDHS showed similar conclusion [5]. This can be partly explained by the notion that recovery of ovarian function was faster among youngsters than older mothers. In addition, younger mothers are less likely to have exposure to health care information about family planning and optimal birth spacing than older mothers.

Although no statistical association was demonstrated, the majority of the study participants reported that short birth interval jeopardizes the health of the mother and the new born child. Similarly, irrespective of their level of birth spacing, the majority of the discussants from all categories underlined that short interbirth intervals are by no means important for the health of the mother, the new born baby, the father, the rest of the children at home, and the overall quality of life of the family:* “if the mother does not get sufficient rest after the preceding birth when she can regain important nutrients, and recovers from physical and psychological health problems the health of the mother herself, and her new born child will be endangered. Yeah, the family will not be stable and peaceful because the disturbance of health condition” (a 35*-*year-old woman from optimal spacing group)*.

When interpreting the finding of the present study, the following limitations should be considered. The source of data for this study was based on the self-report of mothers, and no validation of information was made with any objective sources such as health facility cards except for immunization cards of their children. But respondents were critically informed about the importance of giving accurate information by assuring the confidentiality of their responses and it is logical to assume that biases are less likely in birth interval related events as compared to other sensitive issues. Finally, there could be a recall bias since women were asked for information about events that occurred in the distant past though different life events were used to memorize them.

## 9. Conclusion and Recommendation

Optimal interbirth interval has significant role in reducing fertility and maternal and child mortality. However, birth interval of children varied with different biological and socioeconomic factors of their families. Educational status of the mother, contraceptive utilization, duration of breast-feeding, sex of the preceding child, age during delivery of the last child, and wealth index of respondents were independent predictors of short interbirth interval between the last two live births. Thus, the Ethiopian Ministry of Health together with its stakeholders should strengthen the existing strategies of providing information, education, and communication giving critical attention to women and their husbands to advance their awareness about the importance of modern contraceptive utilization, breast-feeding, and optimal birth spacing. Policy makers should also design new strategies to encourage women to pursue their education to at least primary school level. Moreover, this study considered only a single birth interval and therefore further researches, involving more than two interbirth intervals, should be done to make these findings more informative.

## Supplementary Material

As part of the full length research paper the supplementary material encompasses, location Map of Arba Minch district including all localities, full length English and Amharic (Official language used in Ethiopia) version questionnaire for both qualitative and quantitative studies.
The questionnaire has eight variable categories including Respondents' Identification, Socio-Demographic Characteristics, Birth History, Knowledge of women about Birth spacing, Breast feeding practice , Knowledge and Practice of Modern Contraceptive use, Attitude of respondents towards birth spacing, fertility and family planning, and Household wealth status of respondents.

## Figures and Tables

**Table 1:** Univariate and bivariate analysis of socioeconomic characteristics of child-bearing age mothers in Arba Minch Zuria District, Gamo Gofa Zone, Ethiopia, 2014.

Variables	Cases number (%)	Controls number (%)	Crude Odds Ratio (95% CI)	*p* value
Age at last delivery				
17–24	29 (13.7)	76 (17.9)	0.61 (0.38–1.09)	0.095
25–29	81 (38.2)	181 (42.7)	0.71 (0.44–1.15)	0.163
30–34	63 (29.7)	105 (24.8)	0.95 (0.57–1.60)	0.855
≥35	39 (18.4)	62 (14.6)	1	
Age at first marriage				
12–18	107 (50.5)	226 (53.3)	0.56 (0.24–1.10)	0.173
19–24	94 (44.3)	185 (43.6)	0.60 (0.26–1.39)	0.234
25–29	11 (5.2)	13 (3.1)	1	
Religion				
Protestant	150 (70.8)	284 (67.0)	0.88 (0.157–4.949)	0.887
Orthodox	60 (28.3)	136 (32.1)	1.06 (0.197–5.83)	0.950
Others^*∗*^	2 (0.9)	4 (0.9)	1	
Ethnicity				
Gamo	158 (74.5)	363 (85.6)	2.07 (0.69–6.18)	0.193
Zeysie	50 (23.6)	42 (9.9)	5.65 (1.78–17.92)	0.003
Others^*∗∗*^	4 (1.9)	19 (4.5)	1	
Marital status				
Married	204 (96.32)	414 (97.6)	0.61 (0.24–1.58)	0.315
Single/widowed/divorced	8 (3.8)	10 (2.4)	1	
Educational level of mothers				
No formal education	169 (79.7)	203 (48)	4.28 (2.91–6.29)	0.000
Has formal education	43 (20.3)	221 (52)	1	
Mothers' occupation				
Housewife	195 (92.0)	369 (87.0)	1.81 (0.81–9.79)	0.101
Merchant	9 (4.2)	22 (5.20)	2.18 (0.51–9.36)	0.294
Farmer	5 (2.4)	17 (4.00)	1.57 (0.32–7.66)	0.578
Others^*∗∗∗*^	3 (1.4)	16 (3.8)	1	
Husbands' educational level				
No formal education	132 (64.7)	200 (48.3)	2.01 (1.42–2.83)	0.000
Has formal education	72 (35.3)	214 (51.7)	1	
Husband's occupation				
Farmer	171 (83.8)	342 (82.6)	0.77 (0.421–1.42)	0.403
Daily laborer	15 (7.4)	43 (10.4)	0.53 (0.233–1.22)	0.134
Others^*∗∗∗∗*^	18 (8.8)	29 (7.0)	1	
Wealth index				
Poorest	63 (29.70)	64 (15.10)	5.27 (2.92–9.51)	0.000
Second	48 (22.60)	79 (18.60)	3.25 (1.79–5.91)	0.000
Middle	41 (19.30)	87 (20.50)	2.52 (1.38–4.62)	0.003
Fourth	40 (18.90)	87 (20.50)	2.46 (1.34–4.51)	0.004
Richest	20 (9.40)	107 (25.20)	1	

^*∗*^Hawariya, Ahzab, Yehiwamisikiroch; ^*∗∗*^Welayta, Konso, Amhara, and Oromo; ^*∗∗∗*^daily laborer, student, and employed; ^*∗∗∗∗*^employed, merchant, and fisher.

**Table 2:** Univariate and bivariate analysis of obstetric, breast-feeding, and contraception history of child-bearing age mothers in Arba Minch Zuria District, Gamo Gofa Zone, Ethiopia, 2014.

Variables	Cases number (%)	Controls number (%)	Crude Odds Ratio (95% CI)	*p* value
Number of living children				
0–2	23 (10.8)	48 (11.3)	1	
3-4	99 (46.7)	216 (50.9)	0.957 (0.551–1.66)	0.874
≥5	90 (42.5)	160 (37.7)	1.17 (0.670–2.055)	0.575
Sex of the preceding child				
Female	149 (70.3)	151 (35.6)	4.27 (2.99–6.10)	0.000
Male	63 (29.7)	273 (64.4)	1	
Still birth before last child				
No	207 (97.6)	413 (97.4)	1.10 (0.378–3.215)	0.858
Yes	5 (2.4)	11 (2.6)	1	
Abortion before the last child				
No	206 (97.2)	411 (96.9)	1.09 (0.41–2.90)	0.869
Yes	6 (2.8)	13 (3.1)	1	
Status of the preceding child				
Alive	212 (100)	420 (99)	90.96 (0.000–5.1)	0.504
Dead	0 (0)	4 (0.9)	1	
Previous pregnancy plan				
No	113 (53.3)	125 (29.5)	2.73 (1.94–3.84)	0.000
Yes	99 (46.7)	299 (70.5)	1	
ANC in preceding pregnancy				
No	107 (50.5)	103 (24.3)	3.18 (2.24–4.50)	0.000
Yes	105 (49.5)	331 (75.7)	1	
Place of previous delivery				
Health institution	22 (10.4)	57 (13.4)	0.75 (0.442–1.26)	0.270
Home	190 (89.6)	367 (86.6)	1	
Contraceptive use before last pregnancy				
No	146 (68.9)	141 (33.3)	4.44 (3.12–6.33)	0.000
Yes	66 (31.1)	283 (66.7)	1	
Decision maker about F/P				
Self (mother)	49 (23.10)	86 (20.30)	1	
Both husband and wife	143 (67.5)	294 (69.30)	0.798 (0.423–1.50)	0.485
Husband only	20 (9.40)	44 (10.40)	0.854 (0.57–1.28)	0.442
Husband's attitude towards optimal birth spacing				
Negative	17 (8.30)	24 (5.80)	1	0.178
Positive	187 (91.7)	390 (94.2)	0.648 (0.342–1.22)	
Mothers' attitude towards optimal birth spacing and FP				
Negative	78 (36.80)	158 (37.3)	0.98 (0.696–1.379)	0.908
Positive	134 (63.2)	266 (62.7)	1	
Mother correctly defines optimal birth spacing				
No	55 (25.90)	122 (28.8)	0.87 (0.60–1.258)	0.453
Yes	157 (74.10)	302 (71.2)	1	
Duration of breast-feeding				
0–11 months	22 (10.40)	4 (0.90)	61.95 (20.18–190.18)	0.000
12–23 months	156 (73.6)	37 (8.70)	47.49 (28.76–78.42)	0.000
≥24 months	34 (16.00)	383 (90.30)	1	

**Table 3:** Multivariate analysis of determinant factors among child-bearing age mothers in Arba Minch Zuria District, Gamo Gofa Zone, Ethiopia, 2014.

Variables	Crude Odds Ratio (95% CI)	Adjusted Odds Ratio (95% CI)	*p* value
Educational status of respondents			
No formal education	4.28 (2.91–6.29)	**3.40 **(1.80–6.43)^*∗*^	**0.000**
Has formal education	1	1	
Occupational status of respondents			
Housewife	1.81 (0.81–9.79)	1.00 (0.17–5.86)	1.00
Merchant	2.18 (0.51–9.36)	1.46 (0.16–13.24)	0.737
Farmer	1.57 (0.32–7.66)	2.68 (0.31–23.23)	0.372
Others^♣^	1	1	
Age at delivery of the last child			
17–24	0.61 (0.38–1.09)	0.98 (0.36–2.66)	0.965
25–29	0.71 (0.44–1.15)	0.90 (0.40–2.00)	0.789
30–34	0.95 (0.57–1.60)	2.58 (1.08–6.15)	0.032
≥35	1	1	
Contraceptive use before the last pregnancy			
No	4.44 (3.12–6.33)	**3.01 **(1.68–5.39)^*∗∗*^	**0.000**
Yes	1	**1**	
Sex of the preceding child			
Female	4.27 (2.99–6.10)	**6.79 **(3.65–12.63)^*∗∗*^	**0.000**
Male	1	**1**	
Breast-feeding duration of the preceding child			
<24 months	48.9 (30.02–79.68)	**60.19 **(31.61–114.59)^*∗∗*^	**0.000**
≥24 months	1	**1**	
Place of previous delivery			
Health institution	0.75 (0.442–1.26)	1.53 (0.61–3.80)	0.364
Home	1	1	
Wealth index			
Poorest	5.27 (2.92–9.51)	**14.33 **(4.65–44.15)^*∗∗*^	**0.002**
Second	3.25 (1.79–5.91)	6.46 (2.26–8.48)	0.001
Middle	2.52 (1.38–4.62)	3.98 (1.39–11.38)	0.010
Fourth	2.46 (1.34–4.51)	3.96 (1.41–11.13)	0.009
Richest	1	1	
Previous pregnancy plan			
No	2.73 (1.94–3.84)	1.44 (0.90–2.61)	0.225
Yes	1	1	

^*∗*^Statistically significant at *p* < 0.05; ^*∗∗*^statistically significant at *p* < 0.001;  ^♣^daily laborer, student, and employed.

## Abbreviations

ANC: Antenatal care
AOR: Adjusted Odds Ratio
CSA: Central Statistics Agency
EDHS: Ethiopian Demographic and Health Survey
EPI-INFO: Epidemiological information (statistical software)
FMOH: Federal Ministry of Health
MDGs: Millennium Development Goals
MMR: Maternal mortality ratio
OR: Odds Ratio
SPSS: Statistical Package for Social Sciences
SSA: Sub-Saharan African countries
TFR: Total fertility rate
UNFPA: United Nations Fund for Population Affairs
UNICEF: United Nations International Children's Fund
WHO: World Health Organization.

## References

[1] http://www.un.org/en/development/desa/population/publications/pdf/trends/Concise%20Report%20on%20the%20World%20Population%20Situation%202014/en.pdf.

[2] Federal Democratic Republic of Ethiopia (2010). *Health Sector Development Programme IV 2010/11–2014/15*.

[3] Central Statistical Agency (2007). *Population and Housing Census of Ethiopia: Statistical Report for SNNP Region*.

[4] World Health Organizations (2005). *Make Every Mother and Child Count*.

[5] Central Statistical Agency (2011). *Ethiopia Demographic and Health Survey 2011: Central Statistical Agency Report*.

[6] University of Florida (2008). *Repeat Births and Average Inter birth Intervals Among Medicaid Family Planning Participants*.

[7] John C., Bernstein S., Ezeh A., Faundes A., Glasier A., Innis J. (2006). Family planning: the unfinished agenda. *The Lancet*.

[8] World Health Organization (2005). *Technical Consultation Report on Birth Spacing*.

[9] United States Agency for International Development (USAID) (2005). *Strengthening Family Planning Policies and Programs in Developing Countries*.

[10] Food Policy Research Institute http://www.unsystem.org/SCN/archives/scnnews11/ch12.htm#TopOfPage.

[11] Singh S. N., Singh N., Narendra R. K. (2010). Demographic and socio-economic determinants of birth interval dynamics in manipur: a survival analysis. *The Online Journal of Health and Allied Sciences*.

[12] United Nations (2011). *The Millennium Development Goals Report*.

[13] Abdel-Fattah M., Hifnawy T., El Said T. I., Moharam M. M., Mahmoud M. A. (2007). Determinants of birth spacing among Saudi women. *Journal of Family and Community Medicine*.

[14] Hajian-Tilaki K. O., Asnafi N., Aliakbarnia-Omrani F. (2009). The patterns and determinants of birth intervals in multiparous women in Babol, northern Iran. *The Southeast Asian Journal of Tropical Medicine and Public Health*.

[15] Exavery A., Mrema S., Shamte A. (2012). Levels and correlates of non-adherence to WHO recommended inter-birth intervals in Rufiji, Tanzania. *BMC Pregnancy and Childbirth*.

[16] Rasheed P., Al-Dabal B. (2007). Birth intervals: perceptions and practices among urban based Saudi Arabian women. *International Mediterranean Health Journal*.

[17] Yohannes S., Wondafrash M., Abera M., Girma E. (2011). Duration and determinants of birth interval among women of child bearing age in Southern Ethiopia. *BMC Pregnancy and Childbirth*.

[18] Suwal J. V. (2001). Socio-cultural dynamics of birth intervals in Nepal. *CNAS Journal*.

[19] Rasheed P., Aldabal B. K. (2007). Birth interval: perceptions and practices among urban-based Saudi Arabian women. *Eastern Mediterranean Health Journal*.

[20] Kaharuza F. M., Sabroe S., Basso O. (2001). Choice and chance: determinants of short interpregnancy intervals in Denmark. *Acta Obstetricia et Gynecologica Scandinavica*.

[21] Youssef R. (2005). Duration and determinants of inter-birth interval: community-based survey of women in southern Jordan. *The Eastern Mediterranean Health Journal*.

[22] Asifa K., Muhammad Khalid P. (2012). Determinants of higher order birth intervals in Pakistan. *Journal of Statistics*.

[23] Begna Z., Assegid S., Kassahun W., Gerbaba M. (2013). Determinants of inter birth interval among married women living in rural pastoral communities of southern Ethiopia: a case control study. *BMC Pregnancy and Childbirth*.

[24] Akim J., Mtur I. (1997). The determinants of birth intervals among non-contraceptive user Tanzanian women. *Union for African Population Studies*.

[25] Abdurrahman R., Majid M. (2007). The determinants of birth interval in Ahvaz-Iran: a graphical chain modeling approach. *Journal of Data Science*.

[26] United States agency for International Development (USAID) (2004). *Optimal Birth Spacing: An In-Depth Study of Knowledge, Attitudes and Practices*.

[27] Baschieri A., Hinde A. (2007). The proximate determinants of fertility and birth intervals in Egypt: an application of calendar data. *Demographic Research*.

[28] Odu K., Ogunlade O. (2011). Breastfeeding and child spacing among women in South West Nigeria. *International Journal for Cross-Disciplinary Subjects in Education*.

[29] Rasania S. K., Pathi S., Singh D., Bhalla S., Khandekar J. (2004). Attitude towards birth-spacing: a cross-sectional study among women living in J. J. clusters in Delhi. *Health and Population Perspectives*.

[30] United States agency for International Development http://www.ghtechproject.com/files/South%20Sudan%20Child%20Spacing%20%26%20Family%20Planning-Main%20Report_508%20(secured)_3-6-12.pdf.

[31] Washileh N. (1999). Men's knowledge and attitude towards birth spacing and contraceptive use in Jordan. *International Family Planning Perspective*.

[32] Wikipedia http://en.wikipedia.org/w/index.php?title=Arba_Minch_Zuria&oldid=563667791.

