# Supplementary material for: Determinants of Short Interbirth Interval among Reproductive Age Mothers in Arba Minch District, Ethiopia
Source: Int J Reprod Med. 2016 Apr 27;2016:6072437. doi: 10.1155/2016/6072437 (PMC4863097; doi:10.1155/2016/6072437)
Supplement: Supplementary file 1 — As part of the full length research paper the supplementary material encompasses, location Map of Arba Minch district including all localities, full length English and Amharic (Official language used in Ethiopia) version questionnaire for both qualitative and quantitative studies. The questionnaire has eight variable categories including Respondents' Identification, Socio-Demographic Characteristics, Birth History, Knowledge of women about Birth spacing, Breast feeding practice , Knowledge and Practice of Modern Contraceptive use, Attitude of respondents towards birth spacing, fertility and family planning, and Household wealth status of respondents. [file 6072437.f1.pdf]

11. Annex A

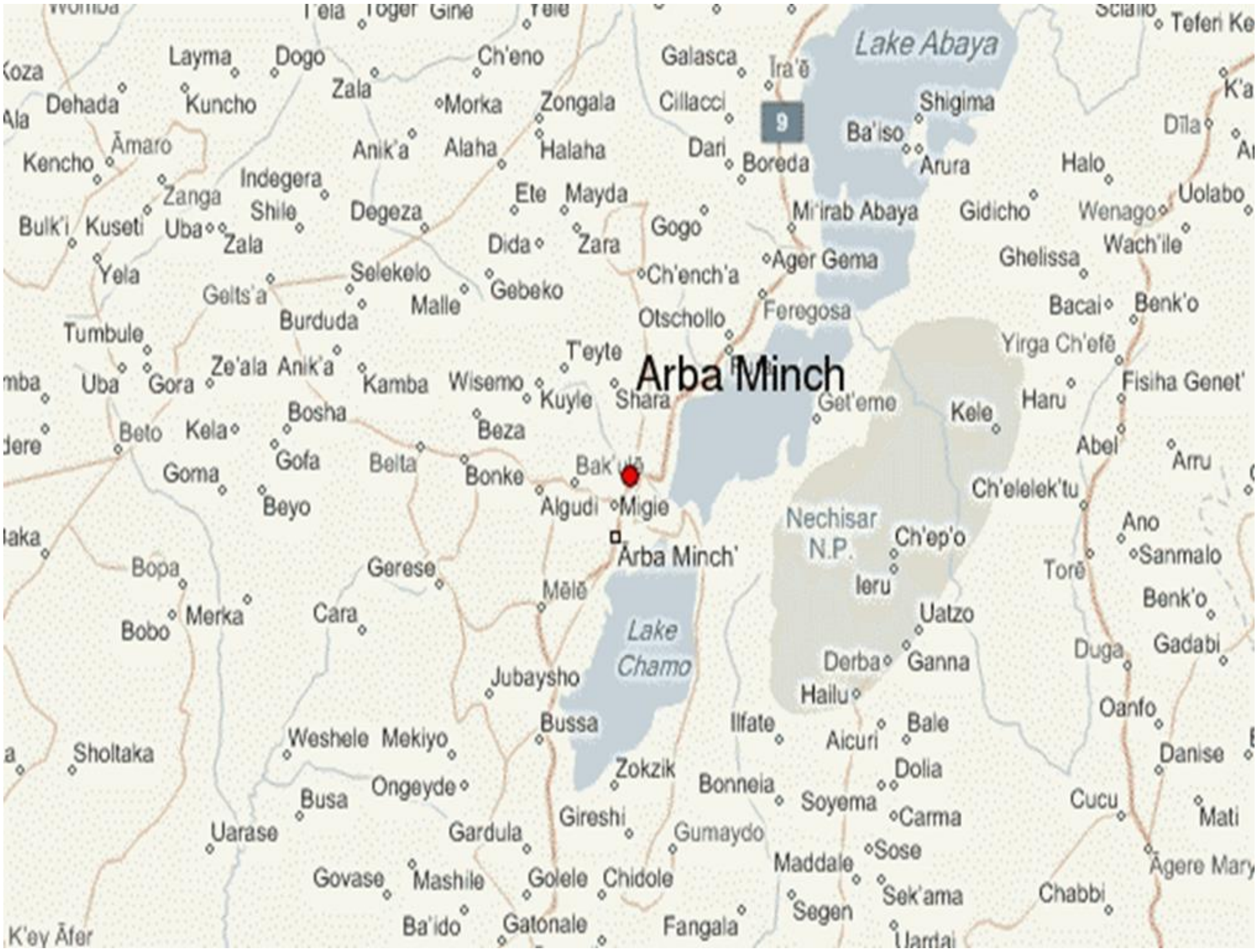

ARBA MINCH'S LOCATION MAP

(Southern Nations, Nationalities, and People's Region, Ethiopia)

Annex B: English version questionnaire

Structured Questionnaire

Arba Minch University College of Medicine and Health Sciences

General information:

Dear Respondent:

My name is ____________. I came from Arbaminch University where I work as a member of the Research team. This questionnaire is prepared to conduct a study on the socio-demographic, socio-economic and biological factors determining inter birth interval among WCBA in this area. You are selected and included in the study as part of the sample population to complete the questionnaire designed by the researcher. The information obtained in this study will be used only for research purposes. The data you will provide is very helpful to achieve the intended objectives of the study. Any information obtained will be kept strictly confidential.

Involvement in this study is optional and in a voluntary basis and you can drop any individual question or the whole questionnaire. But your participation and contribution in the study is very important to come up with important findings which may help local and national health planners to intervene up on the potential problem.

Do you have any opinion regarding this study? Do you agree to participate in this study?

Respondent's Consent

I confirm that I have been given a full explanation of the study and I have understood all about the information given. Therefore, I voluntarily agree to take part in the study.

Signature: _____________________________ Date: ________________________________

**Part I: Respondents' Identification**			
S.N	Questions	Response and Coding	Skip
101	Questionnaire code	------------------	
102	Cluster/Kebele Name	------------------	
103	House Hold Number	------------------	
104	Result of visiting	1.Completed 2.Household absent<br>3.Refused 4.Dwelling destroyed<br>5.Others(specify)---------------	
105	Number of Visits made	1.One 2. Two 3.Three or more	

Part II: Socio-Demographic Characteristics of respondents			
201	How old are you?	_______Years	
202	How is your marital status?	1. Married<br>2. Single<br>3. Divorced<br>4. Widowed<br>5. Separated	
203	At what age did you get married?	_______Years	
204	What is your religion?	1. Orthodox<br>2. Protestant<br>3. Muslim<br>4. Catholic<br>5. Others (specify)_________	
205	What is your ethnicity?	1. Gamo<br>2. Amhara<br>3. Welayta<br>4. Zayse<br>5. Oromo<br>7. Others (specify)________	
206	What is the highest level of school you completed?	1. Illiterate<br>2. Able to read and write<br>3. Elementary (1 - 8)<br>4. Secondary (9 -12)<br>5. More than secondary	
207	What is the highest level of school your husband attended?	1. Illiterate<br>2. Able to read and write<br>3. Elementary (1- 8)<br>4. Secondary (9 -12)<br>5.More than secondary	Skip to
208	What is your primary Occupation?	1. Employed (governmental /Private)<br>2. House wife<br>3. Merchant<br>4. Student<br>5. Farmer<br>6. Daily laborer<br>7. Others (Specify)_________	
209	What is your husband's primary	1. Employed (governmental /Private)<br>2. Merchant	Skip

	Occupation?	3. Student<br>4. Farmer<br>5. Daily laborer<br>6. Others (Specify)___	to
**Part III: Birth History of the respondents**			
301	How many of your own living children do you have? (Probe: Make sure respondent referring to own, biological children.)	Males ____ Females_____	
302	How old were you when you deliver your index child?	_________Years	
303	Did you have antenatal care follow up for your index child?	0.Yes<br>1.No	If 1 go to Q306
304	If your answer for Q303 is yes how many times did you visit ANC clinic?	_______time/s	
305	During ANC follow up did you get any information regarding optimal birth interval?	0.Yes<br>1.No	
306	Where did you deliver your index child?	0.Health facility<br>1.Home	
307	What was the Sex of the index child?	0.Female 1.Male	
308	How was the pregnancy of the index child?	1.Singleton 2.Twins<br>3.Multiple	
309	How was the survival status of the index child?	0. Alive 1. Dead	
310	If died, how old was he/she died?	________Months	
311	Did you have history of still birth b/n the index and last child?	0.Yes 1.No	
312	Did you have history of abortion b/n the index and last child?	0.Yes 1.No	

313	Was the last pregnancy planned?	0.Yes 1.No	
314	At the time you became pregnant with the index child did you want to have more children?	0. Yes<br>1. No	If 1 go to Q318
315	If your answer to Q313 is yes, how many additional children	___________	
316	If your answer to Q313 is yes, was your preference to become pregnant then or wait until later?	0.To become pregnant then<br>1.To wait until later	
317	If your preference was to wait until later, how long did you prefer to wait?	___________	
318	What was the reason to become pregnant then while preferring to wait until later?	____________	
319	In your opinion, how long should the parents wait between one pregnancy and the next?	______________months	
320	Have you talked to your husband about the number of children you would like to have?	0. Yes<br>1. No	
321	Who is the ultimate decision maker regarding birth spacing?	1.Self<br>2.Husband<br>3.Both<br>4.Othere family Members<br>5. Others(Specify)-----------	
322	How was the belief of the husband regarding birth spacing?	0.Supports<br>1.Opposes	
323	How old were you at delivery of the last child?	----------------	
324	Where did you deliver the last child?	0. Health facility<br>1. Home	

325	What was the length of the birth interval since the previous delivery?	_______Months	
**Part IV: Knowledge of women about Birth spacing**			
401	Have you ever heard about optimal birth interval between two consecutive births?	0. Yes<br>1. No	If 1go to Q403
402	If your answer to Q401 is yes, what is the optimum number of years between two consecutive births?	1. Below 3 years<br>2. 3 to 5 years<br>3. Above 5 years<br>4. I don't know	
403	Which birth interval do you think have health advantage?	1. Below 3 years<br>2. 3 to 5 years<br>3. Above 5 years<br>4. I don't know	
404	If your answer to Q403 is 3-5 years, for whom do you think have a health advantages?<br>(More than one answer possible)	1. To the mother<br>2. To the child<br>3.Others(specify)______	
405	Which birth interval has health disadvantages?	1. Below 3 years<br>2. 3 to 5 years<br>3. Above 5 years<br>4. I don't know	
406	If your answer to Q405 is below 3 years, for whom do you think have a health disadvantage?<br>(More than one answer possible)	1. To the mother?<br>2. To the child?	
**Part V: Breast feeding practice of respondents**			

S.N	Questions	Next to Last Child (Response)	Skip
501.	Did you breast feed for your index child?	0. Yes<br>1. No	If 1 go to Q503
502.	If your answer to Q501 is yes, for how long did you breast feed?	______months	
503.	If you stopped breast feeding, what was the reason?	1. Child was old enough<br>2. New pregnancy occurred<br>3. I was sick<br>4. Others(specify)_________	
504.	When do you think breast feeding should stop?	After _______ months.	
**Part VI: Knowledge and Practice of Modern Contraceptive use**			
S.N	Questions	Response and Coding	Skip
601	Do you know any modern female contraceptive method used to limit or avoid pregnancy?	0. Yes<br>1. No	If 1 go to Q603
602	If your answer to Q601 is yes, which methods do you know?<br>(more than one answer possible)	1. Pills<br>2. Injectable<br>3. Condom<br>4. Implants<br>5. IUD<br>6. Others(Specify)---------	
603	Have you been using any of the modern contraceptive methods before you got pregnant for the last child?	0.Yes<br>1.No	if 1 go to Q606
604	If your answer to Q603 is yes, what was the	1. Birth spacing<br>2. Limiting birth	

	purpose?	3.Other(specify)_________	
605	If your answer to Q603 is yes, which modern method/s did you use?	1. Pills<br>2. Injectable<br>3. Condom<br>4. Implant<br>5. IUCD	
606	Are you using any of the modern methods now?	0. Yes 1. No	If 1 go to Q608
607	If yes to question 603 or 606, from where have you got family planning service?	1. Health post 2. Health center<br>3. Hospital 4. Private sector	
608	Did your husband encourage or discourage you to use modern contraception method?	1.Encourage<br>2.Discourage<br>3.Neither<br>4.Don't know	
609	If he discouraged you, what was the reason?	1.Change methods<br>2.Stop using FP<br>3.Wait until we have more children<br>4.Don't know<br>5.Other,___________	
610	If you were not using any contraceptive method to delay or avoid pregnancy, would you tell me the main reason?	1. Desire for more children<br>2. Health problem<br>3. Religious reason<br>4. Cultural reason<br>5. Lack of information about contraception<br>6. FP service not available	

		7. Others /specify/_______	
**Part VII: Attitude of respondents to wards birth spacing, fertility and family planning**			
701	Increasing the number of children affects the quality of health care provided to them	1.Strongly agree<br>2. Agree<br>3.No opinion<br>4.Disagree<br>5.Strongly disagree	
702	Compared with small families large families are less happy	1.Strongly agree<br>2. Agree<br>3.No opinion<br>4.Disagree<br>5.Strongly disagree	
703	Frequent pregnancies may lead to health problems	1.Strongly agree<br>2. Agree<br>3.No opinion<br>4.Disagree<br>5.Strongly disagree	
704	A large number of children may lead to parents tiredness and some psychological problems	1.Strongly agree<br>2. Agree<br>3.No opinion<br>4.Disagree<br>5.Strongly disagree	
705	Having few children may cause a person to feel economically insecure in old age	1.Strongly agree	

		2. Agree<br>3.No opinion<br>4.Disagree<br>5.Strongly disagree	
706	women are as much responsible for planning pregnancies as men	1.Strongly agree<br>2. Agree<br>3.No opinion<br>4.Disagree<br>5.Strongly disagree	
707	Parents with fewer children have better economic status	1.Strongly agree<br>2. Agree<br>3.No opinion<br>4.Disagree<br>5.Strongly disagree	
708	Women should not limit the number of children they should have as it is against the will of God?	1.Strongly agree<br>2. Agree<br>3.No opinion<br>4.Disagree<br>5.Strongly disagree	
709	Having many children is important for the woman to prove her fertility.	1.Strongly agree<br>2. Agree<br>3.No opinion<br>4.Disagree<br>5.Strongly disagree	

710	The family that has all girls should keep having children until they have at least one boy.	1.Strongly agree<br>2. Agree<br>3.No opinion<br>4.Disagree<br>5.Strongly disagree	
711	Budgeting many children can be harmful to the physical and mental health of the mother	1.Strongly agree<br>2. Agree<br>3.No opinion<br>4.Disagree<br>5.Strongly disagree	
712	Having many children can be harmful to the physical and mental health of the father	1.Strongly agree<br>2. Agree<br>3.No opinion<br>4.Disagree<br>5.Strongly disagree	
713	Having many children can be harmful to the health of the new born and the preceding child.	1.Strongly agree<br>2. Agree<br>3.No opinion<br>4.Disagree<br>5.Strongly disagree	
714	If a woman uses modern contraception her satisfaction with sex may decrease	1.Strongly agree<br>2. Agree<br>3.No opinion<br>4.Disagree	

		5.Strongly disagree	
715	Contraception use may cause infertility in women	1.Strongly agree<br>2. Agree<br>3.No opinion<br>4.Disagree<br>5.Strongly disagree	
716	Family planning improves the family's standard of living	1.Strongly agree<br>2. Agree<br>3.No opinion<br>4.Disagree<br>5.Strongly disagree	
717	Family planning gives parents enough time to take care of the needs of their family	1.Strongly agree<br>2. Agree<br>3.No opinion<br>4.Disagree<br>5.Strongly disagree	
**Part VIII: Household wealth status of respondents**			
801	What is the main source of drinking water for the members of your household?	1. Piped water<br>2. Protected well<br>3. unprotected well<br>4. protected spring<br>5. unprotected spring<br>6. surface water (River, pond)<br>7. Others(specify)_______________	
802	Do you have toilet?	0. Yes<br>1. No	
803	Is the toilet private?	0. Yes<br>1.No	
804	What kind of toilet facility do members of	1. Flush toilet	

	your household usually use?	2. Ventilated improved pit latrine (VIP)<br>3. Pit latrine with slab<br>4. Pit latrine without slab/ open pit<br>5. others (specify _____________	
805	Which of the following assets do you have in your household?	1) Electricity<br>2) Watch<br>3) Radio<br>4) Television<br>5) Mobile telephone<br>6) Non-mobile telephone<br>7) Refrigerator?<br>8) Table?<br>9) Chair?<br>10) Sofa<br>11) Bed?<br>12) Cabinet<br>13) Car<br>14) Motorcycle<br>15) Animal-drawn cart<br>16) Bicycle<br>17) Others(specify)____________	
806	What type of fuel do you use for cooking in your household?<br>*(Circle only one answer)*	1) Electricity<br>2) Biogas<br>3) Kerosene<br>4) Charcoal<br>5) Wood<br>6) Animal dung<br>7) Others (specify)_______________	
807	Do have residential home?	0.yes<br>1.No	
808	Number of members per sleeping room?	_________	
809	Do you have separate kitchen room?	0. Yes<br>1. No	
810	What is the material of your house floor? (observation)	1) Ceramic tiles<br>2) Cement/bricks<br>3) Wood	

	Circle only one answer	4) Mud<br>5) Other (specify)_____________	
811	What is the material of the roof of your house (observation)<br>*Circle only one answer*	1) Corrugated iron sheet<br>2) Cement<br>3) Wood<br>4) Plastic sheets<br>5) Thatch/leaf<br>6) Other (specify)______________	
812	What is the material of the walls of your house (observation)<br>*Circle only one answer*	1) Wood with mud<br>2) Wood with Cement<br>3) Stone with cement<br>4) Bricks<br>5) Other[specify]________________	
813	What is the material of the Windows?<br>(Observation)	1.Shutters<br>2.Glass<br>3.Screens<br>5.Others(specify)	
814	What is the primary source of income for this household?<br>*Circle only one answer*	1. Farming, including cash crops<br>2. Livestock<br>3. Employment/salary<br>4. Petty trading (including sale of fire-wood, charcoal, grass)<br>5. Daily labor<br>6.Others(specify)_____________	
815	Does this household own land used for agriculture?	0. Yes<br>1. No	
816	If yes how much land does your household own?	____Hectares	
817	Does this household own any livestock, herds, or farm animals?	0. Yes<br>1. No	
818	How many of the following animals do this household own?<br>[Multiple answer is possible]	1. Cows___ 2. Oxen/bulls_____<br>2. Horses/Donkeys/ Mules_______<br>3. Sheep/Goats____________<br>4. Others(specify)_____________	

Annex C: Amharic version Questionnaire

አርባምንጭ ዩኒቨርሲቲ ህክምናና ጤና ሳይንስ ኮሌጅ

በአርባምንጭ ዙሪያ ወረዳ የእናቶች የአራርቆ መውለድ ባህል የሚገዱ ጉዳዮች ለማጥናት የተዘጋጀ ማጠይቅ

ለተሳታፊዎች የሚነገር አጭር መረጃ

ትውውቅ

ጤና ይስጥልኝ እኔ--------------------እባላለሁ፡፡ በአርባምንጭ ዩኒቨርሲቲ ህክምናና ጤና ሳይንስ ኮሌጅ የጥናት ቡድን አባል ነኝ፡፡ጥናቱ በሳይንሳዊ መንገድ በተመረጡ ቀበሌዎችና ቤቶች ላይ የሚካሄድ ነው፡፡የጥናቱ ዓላማ እናቶች አራርቀው እንዳይወልዱ የሚገዱ ጉዳዮችን ለይቶ የመፍትሄ መንገዶችን ማጠቆም ነው፡፡

ለዚሁ ዓላማ የርስዎን ማህበራዊ ና ስነህዝባዊ፣ የወሊድ ታሪክ፣ ምጣኔሃብታዊ፣በአራርቆ መወለድና ወሊድ መቆጣጠሪያ ዘዴዎች ያሎትን ግንዛቤ ና አመለካከት እንዲሁም ጤና የማጥባት ሁኔታ መረጃ እንሰበስባለን፡፡በመሆኑም ከዚህ ጥናት የሚገኝ መረጃ ሚስጥራዊነቱ የተጠበቀና የጥናቱን ዓላማ ለማሳካት ብቻ የሚውል ሆኖ የሚሰጡት መረጃ መንግስትና ሌሎች የሚመለከታቸዉ ባለድርሻ አካላት የእናቶች የወሊድ መቆጣጠሪያ አጠቃቀምና የአራርቆ መውልድ ባህል ለማሻሻል የመፍትሄ መንገዶችን እንዲቀይሱ ይረዳቸዋል፡፡

በጥናቱ ላይ የሚሳተፉት በፍላጎትዎ ሲሆን በሙሉም ሆነ በከፊል ያለመሳተፍ መብትዎ የተጠበቀ ነዉ፡፡ ጥናቱ ላይ ያለመሳተፍ ዉሳኔዎ የተከበረ ከመሆኑም በላይ ምክጥርዎት እንደሚባክን እርግጠኛ ይሁኑ፡፡

እስካሁን በተነጋገርንባቸዉ ጉዳዮች ላይ ያልገባዎትና ግልፅ ያልሆነ ነገር ካለ ማጠየቅ ይችላሉ፡፡አሁን በጥናቱ ላይ ለመሳተፍ ተስማምተዋል?

አዎን ተስማምቻለሁ-------------- አልተስማማሁም------------- ፈቃደኛ ካለሆኑ ዉሳኔያቸዉን አክብረው በማመስገን ወደ ቀጣዩ ቤት ይሂዱ፡፡

1. ማጠይቁን የሞላው ሰው ስም________________________ፊርማ____________ቀን____________
2. ያረጋገጠው ተቆጣጣሪ ሰው ስም______________________ፊርማ____________ቀን____________

ክፍል I: የጥናቱ ተሳታፊዎች ማለያ

ተ.ቁ	ጥያቄዎች	የመልስ አማራጭ	እለፍ/ፊ
101	የጥያቄ ማህያ ኮድ	------------------	
102	የቀበሌ ስም	------------------	
103	የቤት ቁጥር	------------------	
104	የጉብኝቱ ወጤት	1.ተሟልቷል 2.ቤቱ የለም<br>3.ተሳታፊዋ ፈቃደኛ አይደሉም<br>4.መኖሪያ ቤቱ ፈርሷል<br>5.ሌላ(ይገለፅ) ---------	
105	ቤቱ የተጎበኘበት ብዘት	1.አንድ ጊዜ 2. ሁለት ጊዜ<br>3.ሶስት እና ከዚያ በላይ	
ክፍልII: የተሳታፊዎች ማሰረታዊ ሚጃ			
201	ስንት አመትዎ ነው?	_______ዓመት	
202	የጋብቻ ሁኔታ	1. ያገባች<br>2. ያላገባች<br>3. የተፋታች<br>4. ባል የሞተባት<br>5. ተለያይተው የሚኖሩ	
203	በስንት አመትዎ ትዳር ማሰረቱ ?	_______ዓመት	
204	የምን እምነት ተከታይ ነዎት ?	1. ኦርቶዶክስ<br>2. ፕሮቴስታንት<br>3. ሙስሊም<br>4. ካቶሊክ<br>5. ሌላ(ይገለፅ)__________	
205	ብሄርዎ ምንድነው?	1. ጋሞ<br>2. አማራ<br>3. ወላይታ<br>4. ዘይሴ<br>5. ኦሮሞ<br>7. ሌላ(ይገለፅ) _________	
206	የትምህርት ደረጃ	1. ያልተማሂች<br>2. ማንበብና ማፍ የምትችል<br>3. ከ1-8ኛ ክፍል<br>4.ከ9-12ኛ ክፍል<br>5. ከ2ኛ ደረጃ በላይ	
207	የባለቤትዎ የትምህርት ደረጃ	1. ያልተማሂ<br>2. ማንበብና ማፍ የሚችል<br>3. ከ1-8ኛ ክፍል<br>4. ከ9-12ኛ ክፍል<br>5.ከ2ኛ ደረጃ በላይ	
208	የስራ ሁኔታ	1. ተቀጣሪ (የመንግስት/የግል)	

		2. የቤት እመቤት<br>3. ነጋዴ<br>4. ተማሪ<br>5. አርሶ አደር<br>6. የቀን ሰራተኛ<br>7. ሌላ (ይገለፅ) ___________	
209	የባለቤትዎ የስራ ሁኔታ	1. ተቀጣሪ (የመንግስት/የግል)<br>2. ነጋዴ<br>3. ተማሪ<br>4. አርሶ አደር<br>5. የቀን ሰራተኛ<br>6. ሌላ (ይገለፅ)____	
**ክፍል III: የጥናቱ ተሳታፊዎች የወሊድ ታሪክ**			
301	በህይዎት ያሉ ስንት ልጆች አሉዎት?	ወንድ _____ሴት______	
302	የመጀመሪያ ልጅዎን የወለዱት በስንት አመትዎ ነው?	_______ዓመት	
303	የመጀመሪያ ልጅዎን እንዳረገዙ የእርግዝና ክትትል አድርገው ነበር ?	0.አዎ<br>1.አላደረኩም	መልሳቸው 1 ከሆነ 306
304	ለጥያቄ ቁጥር 303 መልስዎ አዎ ከሆነ ስንት ጊዜ የእርግዝና ክትትል አድርገዋል?	______ጊዜ	
305	የእርግዝና ክትትል በሚያደርጉበት ወቅት አራርቆ ስለመወለድ ትምህርት አግኝተዋል?	0.አዎ<br>1.አላገኘሁም	
306	የመጀመሪያ ልጅዎን የት ነው የወለዱት ?	0. ጤና ተቋም<br>1.ቤት	
307	የመጀመሪያ ልጅዎ ፆታ ምን ነበር?	0.ሴት 1.ወንድ	
308	በመጀመሪያ እርግዝናዎ ስንት ልጆችን ተገላገሉ/ወለዱ?	1.ነጠላ/አንድ 2.መንታ<br>3.ብዙ	
309	የመጀመሪያ ልጅዎ በህይዎት አለ?	0. በህይዎት አለ 1.ሞተዋል	
310	መልስዎ ሞተ ከሆነ፣ በስንት ጊዜው ሞተ?	_______ወር	
311	በሚጨርሰዉ ከሰብሬት ባለው ልጅዎት መሀል ሞቶ የተወለደ ህፃን ነበረዎት?	0.አዎ 1. አልነበረኝም	
312	በሚጨርሰዉ ከሰብሬት ባለው ልጅዎት መሀል ወርጃ አጋጥሞዎት ነበር?	0.አዎ<br>1. አላጋጠመኝም	
313	ከአሁን በፊት ያለው እርግዝና የታቀደ ነበር?	0.አዎ<br>1.አይደለም	

314	የመጀመሪያ ልጅዎን ነፍሰጡር በነበሩበት ወቅት ተጨማሪ ልጅ የመዉለድ ፍላጎት ነበረዎት?	0. አዎ<br>1. አልነበረኝም	መልስዎ 1 ወደ 319
315	ለጥያቄ ቁጥር 314 መልስዎ አዎ ከሆነ ምን ያህል ልጅ እንዲኖርዎት ነበር የፈለጉት?	________	
316	ለጥያቄ ቁጥር 314 መልስዎ አዎ ከሆነ ምርጫዎ ወዲያዉኑ ማርገዝ ነበር ወይስ የመቆየት እቅድ ነበረዎት ?	0. ወዲያዉኑ ማርገዝ<br>1.እስከተወሰነ ጊዜ ድረስ መቆየት	
317	ልጅ ሳይወልዱ የመቆየት እቅድ ከነበረዎት ለምን ያህል ጊዜ ነበር መቆየት የፈለጉት ?	_______ወር	
318	ልጅ ሳይወልዱ የመቆየት እቅድ ከነበረዎት ለምን ነበር ቶሎ ለማርገዝ የወሰኑት?	________	
319	በእርስዎ አስተያየት ወላጆች አንድ ልጅ ከወለዱ በሃላ ሌላ ልጅ ለመዉለድ ምን ያህል ጊዜ መቆየት አለባቸው?	_______ወር	
320	ምን ያህል ልጆች ሊኖርዎት እንደሚፈልጉ ከባለቤትዎ ጋ ተነጋግረው ያዉቃሉ?	0. አዎ<br>1.አላወቅም	
321	እናንተ ቤተሰብ ወስጥ ስለ አራርቆ መዉለድ ወሳኙ ማነ ው ?	1.እኔ<br>2.ባለቤቴ<br>3.እኔና ባለቤቴ<br>4.ሌላ የቤተሰብ አባል<br>5. ሌላ(ይገለፅ)-----------	
322	ባለቤትዎ ስለ አራርቆ መዉለድ ያላቸው አቋም ምን ነበር ?	0.ይደግፋሉ<br>1.ይቃወሟሉ	
323	የመጨረሻ ልጅዎን ሲወልዱ ስንት አመትዎ ነበር?	----------------	
324	የመጨረሻ ልጅዎን የት ነበር የወለዱት ?	0. ጤና ተቋም<br>1. ቤት	
325	በመጨረሻ ልጅዎና በእሱ/ሳ ታናሽ ምን ያህል የእድሜ ልዩነት አለ ?	________ወር	
**ክፍል IV: እናቶች ስለ አራርቆ መዉለድ ያላቸዉን እዉቀት በተመለከተ**			
401	በሁለት እርግዝና መካከል ምን ያህል የእድሜ ልዩነት መኖር እንዳለበት ሰምተው	0. አዎ	If መልስዎ 1

	ያወቃሉ?	1. ሰምቼ አላወቅም	ወደ 403
402	ለጥያቄ ቁጥር 401 መልስዎ አዎ ከሆነ ተመጣጣኝ የሆነው እድሜ ስንት ነው ብለው ያስባሉ ?	5. ከሶስት አመት በታች<br>6. ከ3-5 አመት<br>7. ከ5 አመት በላይ<br>8. አላወቀውም	
403	ልጆች በምን ያህል እድሜ ተራርቀው ቢወለዱ ለጤና ጥሩ ነው ብለው ያስባሉ?	1. ከሶስት አመት በታች<br>2. ከ3-5 አመት<br>3. ከ5 አመት በላይ<br>4. አላወቀውም	መልስ ከ 2 ውጭ ከሆነ ወደ 405
404	ለጥያቄ ቁጥር 403 መልስዎ 3-5 አመት ከሆነ የጤና ጠቀሜታው ለማ ው ነው?<br>(ከአንድ በላይ መልስ ይቻላል)	1. ለእናት<br>2. ለህፃኑ<br>3.ሌላ(ይገለፅ) _______	
405	ልጆች በየትኛው የእድሜ ልዩነት ቢወለዱ ለጤና መጥፎ ነው ብለው ያስባሉ?	1. ከሶስት አመት በታች<br>2. ከ3-5 አመት<br>3. ከ5 አመት በላይ<br>4. አላወቀውም	መልስዎ ከ 1 ውጭ ከሆነ ወደ 501
406	ለጥያቄ ቁጥር 405 መልስዎ ከ3 አመት በታች ከሆነ ለጤና መጥፎ የሚሆነው ለማን ነው?<br>(ከአንድ በላይ መልስ ይቻላል)	1.ለእናት<br>2. ለህፃን/ለልጅ<br>3. ሌላ(ይገለፅ)--------------	
**ክፍልV: ጡት ማጥባትን በተመለከተ**			
ተ.ቁ	ጥያቄዎች	የመልስ አማራጮች	ይለፍ
501.	የመጀመሪያ ልጅዎን ጡት አጥብተዋል?	0. አዎ<br>1. ጡት አጥብቼ አላወቅም	መልስዎ 1ከሆነ ወደ 503
502.	ለጥያቄ ቁጥር 501 መልስዎ አዎ ከሆነ ለምን ያህል ጊዜ ነው ያጠቡት ?	_______ወር	
503.	ጡት ማጥባት ለማቆም ምክነያትዎ ምን ነበር ?	1. ልጁ ትልቅስለሆነ<br>2. ስላረገዝኩ ነበር<br>3. ታምሜ ስለነበር ነው	

		4. ሌላ (ይገለፅ) __________	
504.	ጠቅ ማጥባት መቆም ያለበት መቸ ነው ብለው ያስባሉ?	ከ ___ወር በኋላ.	
**ክፍል VI: ዘመናዊ የወሊድ ማካላከያ እውቀትና አጠቃቀምን በተመለከተ**			
ተ.ቁ	ጥያቄ	የመልስ አማራጭ	ይለፍ
601	ዘመናዊ የሆነ የወሊድ ማካላከያ ያወቃሉ?	0. አዎ<br>1. አላወቅም	መልስዎ1ከሆነ ወደ 603
602	ለጥያቄ ቁጥር 601 መልስዎ አዎ ከሆነ የትኛውን አይነት የወሊድ ማካላከያ ዘዴ ያወቃሉ?<br>(ከአንድ በላይ መልስ ይቻላል)	2. በአፍ የሚዋጥ እንክብል<br>3. በመርፌ የሚሰጥ/የሚወሰድ<br>4. ኮንዶም<br>5. ክንድ ላይ የሚቀበር<br>6. ማህፀን ወስጥ የሚገባ/የሚቀመጥ<br>7. ሌላ (ይገለፅ) ---------	
603	የመጨረሻ ልጅዎን ከመርገዝዎ በፊት ዘመናዊ የወለድ ማካለከያ ይጠቀሙ ነበር?	0. አዎ<br>1. አልጠቀምም	መልስዎ1ወደ606
604	ለጥያቄ ቁጥር 603 መልስዎ አዎ ከሆነ ለምን ነበር የሚጠቀሙት?	1. አራርቆ ለመወለድ<br>2. መወለድ ለማቆም<br>3. ሌላ (ይገለፅ)___________	
605	ለጥያቄ ቁጥር 603 መልስዎ አዎ ከሆነ የትኛውን ዘመናዊ የወሊድ ማካላከያ ነበር የሚጠቀሙት?	6. እንክብል<br>7. በመርፌ የሚወሰድ<br>8. ኮንዶም<br>9. ክንድ ላይ የሚቀበር<br>10. ማህጸን ወስጥ የሚገባ/የሚቀመጥ	
606	በአሁኑ ሰአት ዘመናዊ የወሊድ ማካላከያ ዘዴ ይጠቀማሉ?	0. አዎ 1. አልጠቀምም	መልስዎ 1 ወደ 608
607	ለጥያቄ ቁጥር 603/606 መልስዎ አዎ ከሆነ ከየት አግኝተው ነው የሚጠቀሙት ?	1. ጤና ኬላ 2. ጤና ጣቢያ<br>3. ሆስፒታል 4. ከግል ጤና ተቋም	
608	ባለቤትዎ ዘመናዊ የወሊድ ማካላከያ እንዲጠቀሙ ያበረታቱዎታል ወይስ አያበረታቱዎትም?	1.ያበረታታኛል<br>2.አያበረታታኝም/ያጣጥልብኛል<br>3.ምንም አይልም<br>4.አላወቅም	መልስ ከ 2 ውጭ ከሆነ ወደ 610
609	የሚያበረታቱዎት ከሆነ ምክንያቱ ምን ነበር ?	1.የማካላከያ ዘዴ እንድቀይር ስለሚፈልግ	

		2.የወሊድ መከላከያ እንዳቆም<br>3.ብዙ ልጆች እንዲኖሩን ስለሚፈልግ<br>4.አላወቀውም<br>5.ሌላ (ይገለፅ) ____________	
610	የወሊድ መከላከያ የሚጠቀሙ ከሆነ ምክንያቱዋን ሊነግሩኝ ይችላሉ?	8. ብዙ ልጆች መውለድስለምፈልግ<br>9. የጤና ችግር ስላለብኝ<br>10. ሐይማኖቴ ስለሚፈቅድ<br>11. በባህላችን ስለሚፈቀድ<br>12. ስለወሊድ መከላከያ ሚሺኛ ስለሌለኝ<br>13. የቤተሰብ ምጣኔ አገልግሎት ስለሌለ<br>14. ሌላ (ይገለፅ) ________	
**ክፍል VII: አራርቆ መውለድና የቤተሰብ ምጣኔን በተመለከተ ተሳታፊዎች ያላቸውን አመለካከት ለማወቅ የተዘጋጀ**			
701	የልጆች ቁጥር መብዛት ለልጆች የሚሰጠውን የጤና አገልግሎት ጥራት ይቀንሰዋል?	1.በጣም እስማማለሁ<br>2. እስማማለሁ<br>3.አስተያየት የለኝም<br>4.አልስማማም<br>5.በጣም አልስማማም	
702	ብዙ ቤተሰብ ያላቸው ሰዎች ትንሽ ቤተሰብ ካላቸው ሲወዳደሩ ደስተኞች አይደሉም ?	1.በጣም እስማማለሁ<br>2. እስማማለሁ<br>3.አስተያየት የለኝም<br>4.አልስማማም<br>5.በጣም አልስማማም	
703	ቶሎቶሎ ማርገዝ ለጤና ችግር ሊያጋልጥ ይችላል ?	1.በጣም እስማማለሁ<br>2. እስማማለሁ<br>3.አስተያየት የለኝም<br>4.አልስማማም<br>5.በጣም አልስማማም	
704	ብዙ ልጆች መኖራቸው ወላጆች ላይ	1.በጣም እስማማለሁ	

	የስነልቦና ችግር ሊያስከትል ይችላል?	2. እስማማለሁ<br>3.አስተያየት የለኝም<br>4.አልስማማም<br>5.በጣም አልስማማም	
705	ትንሽ/ጥቂት ልጆች መኖራቸው ወላጆች በሚያረጁበት ጊዜ ኢኮኖሚያቸው የተረጋጋ እንዳይሆን ሊያደርግ ይችላል?	1.በጣም እስማማለሁ<br>2. እስማማለሁ<br>3.አስተያየት የለኝም<br>4.አልስማማም<br>5.በጣም አልስማማም	
706	ሴቶች ቤተሰብን ከመጣን አካያ ከወንዶች እኩል ድርሻ አላቸው?	1.በጣም እስማማለሁ<br>2. እስማማለሁ<br>3.አስተያየት የለኝም<br>4.አልስማማም<br>5.በጣም አልስማማም	
707	ጥቂት ልጆች ያላቸው ቤተሰቦች የኢኮኖሚ ደረጃቸው የተሻለ ነው?	1.በጣም እስማማለሁ<br>2. እስማማለሁ<br>3.አስተያየት የለኝም<br>4.አልስማማም<br>5.በጣም አልስማማም	
708	ከእግዚአብሄር ፈቃድ ውጭ ስለሆነ ሴቶች የሚወልዷቸውን ልጆች መጣን የለባቸውም ?	1.በጣም እስማማለሁ<br>2. እስማማለሁ<br>3.አስተያየት የለኝም<br>4.አልስማማም<br>5.በጣም አልስማማም	
709	ብዙ ልጆች መኖራቸው የሴቶችን ወላደነት/የመወለድ አቅም ለሚረጋገጥ አስፈላጊ ነው/ይጠቅማል ?	1.በጣም እስማማለሁ<br>2. እስማማለሁ<br>3.አስተያየት የለኝም<br>4.አልስማማም	

		5.በጣም አልስማም	
710	ሴት ልጆች ብቻ ያላቸው ቤተሰቦች ወንድ ልጅ እስኪያገኙ ድረስ መውለድ አለባቸው ?	1.በጣም እስማማለሁ<br>2. እስማማለሁ<br>3.አስተያየት የለኝም<br>4.አልስማም<br>5.በጣም አልስማም	
711	ብዙ ልጆች መኖራቸው ለእናት አካላዊና ስነልቦናዊ ጠና ችግር ሊዳርግ ይችላል?	1.በጣም እስማማለሁ<br>2. እስማማለሁ<br>3.አስተያየት የለኝም<br>4.አልስማም<br>5.በጣም አልስማም	
712	ብዙ ልጆች መኖራቸው ለአባት አካላዊና ስነልቦናዊ ጠና ችግር ሊዳርግ ይችላል?	1.በጣም እስማማለሁ<br>2. እስማማለሁ<br>3.አስተያየት የለኝም<br>4.አልስማም<br>5.በጣም አልስማም	
713	ብዙ ልጆች መኖራቸው ለሚወለዱት ልጆችና ለተወለዱት ልጆች የጠና ችግር ሊያመጣ ይችላል?	1.በጣም እስማማለሁ<br>2. እስማማለሁ<br>3.አስተያየት የለኝም<br>4.አልስማም<br>5.በጣም አልስማም	
714	ዘመናዊ የወሊድ መከላከያ የሚጠቀሙ ሴቶች የወሲብ እርካታቸው ሊቀንስ ይችላል?	1.በጣም እስማማለሁ<br>2. እስማማለሁ<br>3.አስተያየት የለኝም<br>4.አልስማም<br>5.በጣም አልስማም	
715	የወሊድ መከላከያ መጠቀም ለመካንነት ሊዳርግ ይችላል?	1.በጣም እስማማለሁ<br>2. እስማማለሁ	

		3.አስተያየት የለኝም<br>4.አልስማም<br>5.በጣም አልስማም	
716	የወሊድ መከላከያ ማጠቀም የቤተሰብ የኑሮ ደረጃ እንዲሻሻል ይረዳል?	1.በጣም እስማማለሁ<br>2. እስማማለሁ<br>3.አስተያየት የለኝም<br>4.አልስማም<br>5.በጣም አልስማም	
717	የቤተሰብ ምጣኔ ማጠቀም ለወላጆች በቂ ጊዜ እንዲያገኙና ለቤተሰባቸው ጥሩ እንክብካቤ እንዲያደርጉ ያግዛቸዋል?	1.በጣም እስማማለሁ<br>2. እስማማለሁ<br>3.አስተያየት የለኝም<br>4.አልስማም<br>5.በጣም አልስማም	
**ክፍል VIII:** የቤተሰብ የኑሮ ደረጃን በተመለከተ			
801	የቤተሰባችሁ አባላት የመጠጥ ውሃ ከየት ነው የሚያገኙት?	8. የቧንቧ ውሃ<br>9. ከተከለለ የጉድጓድ ውሃ<br>10. ካልተከለለ የጉድጓድ ውሃ<br>11. ከተከለለ ምንጭ<br>12. ካልተከለለ ምንጭ<br>13. የኩሬ ወይም የወንዝ ውሃ<br>14. ሌላ (ይገለፅ)________________	
802	ሽንት ቤት አላችሁ ?	2. አዎ<br>3. የለንም	መልስ 0 ወደ
803	ሽንት ቤቱ የራሳችሁ ነው ?	0. አዎ<br>1.አይደለም	
804	ምን አይነት ሽንት ቤት ነው የምትጠቀሙት?	6. ፍላሽ ቶይለት<br>7. ቬንትሌትድ ኢምፕሩቭድ ፒት ላትሪን<br>8. በስላብ የተሰራ ፒት ላትሪነ<br>9. ስላብ የሌለው ፒት ላትሪን/ ኦፕን ፒት<br>10. ሌላ (ይገለፅ) ________________	
805	እዚህ ከተጠቀሱት ወስጥ የትኛው በናንተ ቤት ወስጥ አለ ?	18) ኤሌክትሪክ<br>19) ሰዓት<br>20) ሬዲዮ<br>21) ቴሌቪዥን<br>22) የሞባይል ስልክ<br>23) የቤት ስልክ<br>24) ፍሪጅ<br>25) ጠረጴዛ<br>26) ወንበር<br>27) ሶፋ	

		28) አልጋ<br>29) ብፌ<br>30) መኪና<br>31) ሞተር ሳይክል<br>32) ጋሪ<br>33) ብስክሌት<br>34) ሌላ (ይገለፅ)_______________	
806	ምግብ ለማብሰያ የምትጠቀሙት ምንድነው (አንዱን ብቻ ያክብቡ) ?	8) ኤሌክትሪክ<br>9) ባዮጋዝ<br>10) ነጭ ጋዝ<br>11) ከሰል<br>12) እንጨት<br>13) ኩበት<br>14) ሌላ(ይገለፅ)____________________	
807	መኖሪያ ቤት አላችሁ	0.አዎ<br>1.የለንም	
808	በአንድ የሚደሪያ ክፍል ወስጥ ስንት ሰው ነው የሚተኛው/የሚያድረው?	___________	
809	ራሱን የቻለ የምግብ የማበሰያ ክፍል አላችሁ	2. አዎ<br>3. የለንም	
810	ቤቱ ከምንድነው የተሰራው (እባክዎን ይመልከቱት) ?<br>*አንዱን ብቻ ያክብቡ*	6) ከሴራሚክስ<br>7) ከጡብ<br>8) ከእንጭት<br>9) ከጭቃ<br>10) ሌላ (ይገለፅ)_________________	
811	የቤቱ ጣራ ከምንድነው የተሰራው (እባክዎን ይመልከቱት) ?<br>*አንዱን ብቻ ያክብቡ*	7) ከቆርቆሮ<br>8) ከሲሚንቶ<br>9) ከእንጨት<br>10) ከፕላሰቲክ<br>11) ከቅጠል<br>12) ሌላ (ይገለፅ)______________	
812	የቤቱ ወለል ከምንድነው የተሰራው (እባክዎን ይመልከቱት) ?<br>*አንዱን ብቻ ያክብቡ*	6) ከጭቃና ከእንጨት<br>7) ከሲሚንቶና እንጨት<br>8) ከድንጋይ እና ሲሚንቶ<br>9) ከጡብ<br>10) ሌላ (ይገለፅ)_____________	
813	የቤታችሁ ማኮት ከምንድ ነው የተሰራው	1.ከጣወላ የተሰራ ማዝግያ<br>2.ማስተዋት<br>3.ማረጃ አይነት<br>5.ሌላ(ይገለፅ)-------------------	
814	ዋናው የቤተሰባችሁ የገቢ ምንጭ ምንድነው?	1. እርሻ<br>2. እንስሳት እርባታ	

	አንድ ሜለስ ብቻ ያከብቡ	3. የቅጥር ደመወዝ<br>4. ጥቃቅን ንግድ<br>5. የቀን ስራ<br>6.ሌላ (ይገለፅ)________________	
815	የእርሻ መሬት አላችሁ?	2. አዎ<br>3. የለንም	
816	ምን ያህል ሄክታር መሬት አላችሁ?	_____ሄክታር	
817	የቤት እንስሳት አላችሁ?	8. አዎ<br>9. የለንም	
818	ምን ያህል የሚከተሉት የቤት እንስሳት በቤት ወስጥ ይገኛሉ?<br>[ከአንድ በላይ ሜለስ መሜለስ ይቻላል]	5. ላም___________<br>6. በሬ_______________<br>7. ፈረስ/አህያ/ በቅሎ________<br>8. በግ/ፍየል_______________<br>9. ሌላ (ይገለፅ)________________	

Annex D: English version Focus group discussion guide

Introduction

First of all I would like to extend my gratitude to all of you for being willing to come here and participate on this discussion. My name is------------- and my colleagues' name is-----------We came from Arba Minch University to conduct a research on factors contributing to inter birth interval among mothers. Thus, the purpose of this group discussion is to assess your overall knowledge and attitude about family planning and birth spacing.

For the sake of accuracy and efficiency, we will take notes and tape record, unless any one has any objections. In this group everybody should feel free to talk. Each and every opinion is important and wanted. In this group there are no rights or wrong answers.

Even though your participation is important for this study, you have the right to refuse to answer any questions and to end the discussion at any time if you find it necessary to do so.

Location of Discussion----------------------------------

Date of Discussion----------------Time started-------------Timed finished----------------

Moderator’s Name---

Assistant Moderator (note taker’s) name---

Number of Participants---------------------

I. Introduction

At this point, we would like to ask you to introduce yourself to the rest of the group. Let us start with the research team and each of you will tell me your name, how long you have lived in this area and your job.

Perceived Knowledge of women about birth spacing and family planning

1. Have you ever heard about the word birth spacing? Probe
2. Have you ever heard about optimal birth spacing between two consecutive live births?
3. What does short birth interval mean to you?
4. How do you explain the advantage and disadvantage of short birth spacing to the family?
5. How do you explain the advantage and disadvantage of optimal birth spacing to the family?
6. Who is the ultimate decision maker about your family planning utilization and birth spacing practice?
7. What are the reasons for having short birth intervals
8. Have you ever heard of family planning?
9. What are the methods used for delaying or avoiding pregnancy?
10. Have you been using any of the modern contraceptive methods? If not why?
11. Explain how your husband, relatives and your religion affect your decision to utilize contraceptive method and birth spacing.

12. Do you have any more issues, questions, comments that you want to add? Thank you!

Annex E: Amharic version Focus group discussion guide for mothers

I. <u>ለእናቶች እና አባቶች የተዘጋጁ የመወያያ ጥያቄዎች</u>

ውይይት የተደረገበት ቀበሌ ስም----------------------ውይይት የተካሄደበት ቀን----------------
ለውይይት የወሰደበት ግዜ------------------------የአወያይ ስም--------------------------------------
የአወያይ አጋዥ ስም-------------------------------------የተወያዮች ብዛት------------------------

የተወያይ ኮድ	እድሜ	በማህበረሰቡ ያላት አስተዋፅኦ	የትምህርት ደረጃ	ስራ	የልጆች ቢዛት

1. በእናንተ አስተሳሰብ አራርቆ ልጆች መውለድ ማለት ምን ማለት ነው?
2. በእናንተ አመለካከት ልጆች በምን ያህል ግዜ ሉይነት ሲወለዱ ነው ጤነኛ/ጥሩ የሚባለው?
3. በእናንተ አመለካከት ልጆች በምን ያህል ግዜ ሉይነት ሲወለዱ ነው አጭር የሚባለው?
4. በእናንተ አመለካከት ልጆች በአጭር ግዜ(ከሰወስት ዓመት በታች) ሉይነት ቢወለዱ ጉዳቱ ምንድ ነው?
5. በእናንተ አመለካከት ልጆች በሶስት እና ከዚያ በላይ የግዜ ሉይነት ቢወለዱ ጉዳቱ ምንድ ነው?
6. ወላጆች በአጭር ግዜ ሉይነት እንዲወልዱ የሚያደርጉዋቸው ምክንያቶች ምንድን ናቸው?
7. በቤታቹው ውስጥ ልጆች በምን ያህል ግዜ ሉይነት መወለድ እንዳለባቸው የመጨረሻ ውሳኔ የሚሰጠው ማነው?
8. ስለ የቤተ ሰብ ምጣኔ ሰመታችው ታውቃላችው?
9. ወሊድ ለመቆጣጠር የምንጠቀምባቸው ዘመናዊ ዘዴዎች ሊነግሩኝ ይችላሉ?
10. የመጨረሻው ልጅዎ ከማርገዝዎ በፊት ዘመናዊ የወሊድ መቆጣጠሪያ ዘዴ ተጠቅመው ነበር? ካልተጠቀሙ ለምን?

11. አራርቆ በመውለድ እና ዘመናዊ የወሊድ መቆጣጠሪያ ዘዴ በመጠቀም ዙሪያ የባለ ቤትዎ፣ ቤተሰዎችዎ፣ ሀይማኖትዎ እነዲሁም የአከባቢው ባህል ተፅእኖ ምን ይመስላል?

12. በመጨረሻ ጥያቄ ወይም አስተያየት ካላችሁ? አመሰግናለሁ፡፡

Declaration

We, the under signed hereby, declare that this research is our original work and all sources of material used for this work and all the people and institutions that gave support have been duly acknowledged.

Principal investigator: Desta Hailu (BSc, MSc) Signature: ------------ Date: ------------------

Co-Investigator: Teklemariam Gultie (BSc, MSc) Signature: -------------- Date: -----------------

AMU, CMHS Research coordinator:

Name--- signature--------------------- Date: --------------------

AMU, Research directorate director:

Name--- signature--------------------- Date: --------------------
